# Observing separate spin and charge Fermi seas in a strongly correlated one-dimensional conductor

**DOI:** 10.1126/sciadv.abm2781

**Published:** 2022-06-17

**Authors:** Pedro M. T. Vianez, Yiqing Jin, María Moreno, Ankita S. Anirban, Anne Anthore, Wooi Kiat Tan, Jonathan P. Griffiths, Ian Farrer, David A. Ritchie, Andrew J. Schofield, Oleksandr Tsyplyatyev, Christopher J. B. Ford

**Affiliations:** 1 Department of Physics, Cavendish Laboratory, University of Cambridge, Cambridge CB3 0HE, UK.; 2 Departamento de Física Aplicada, Universidad de Salamanca, Plaza de la Merced s/n, 37008 Salamanca, Spain.; 3 Université de Paris, C2N, 91120 Palaiseau, France.; 4 Department of Electronic and Electrical Engineering, University of Sheffield, 3 Solly Street, Sheffield S1 4DE, UK.; 5 Department of Physics, Swansea University, Vivian Tower, Singleton Park, Swansea SA2 8PP, UK.; 6 Department of Physics, Lancaster University, Lancaster LA1 4YB, UK.; 7 Institut für Theoretische Physik, Universität Frankfurt, Max-von-Laue Straße 1, 60438 Frankfurt, Germany.

## Abstract

An electron is usually considered to have only one form of kinetic energy, but could it have more, for its spin and charge, by exciting other electrons? In one dimension (1D), the physics of interacting electrons is captured well at low energies by the Tomonaga-Luttinger model, yet little has been observed experimentally beyond this linear regime. Here, we report on measurements of many-body modes in 1D gated wires using tunneling spectroscopy. We observe two parabolic dispersions, indicative of separate Fermi seas at high energies, associated with spin and charge excitations, together with the emergence of two additional 1D “replica” modes that strengthen with decreasing wire length. The interaction strength is varied by changing the amount of 1D intersubband screening by more than 45%. Our findings not only demonstrate the existence of spin-charge separation in the whole energy band outside the low-energy limit of the Tomonaga-Luttinger model but also set a constraint on the validity of the newer nonlinear Tomonaga-Luttinger theory.

## Teaser

Interacting electrons in one dimension occupy two, rather than the usual one, Fermi seas for their spin and charge.

## INTRODUCTION

Many-body systems cannot be explained by studying their individual components, with interactions often giving rise to collective excitations from which an array of previously unknown quasiparticles starts to emerge. This is particularly notable in one dimension (1D), as here, geometrical confinement alone imposes strong correlations in the presence of any interactions, leading to well-known non–Fermi liquid phenomena such as spin-charge separation (*1*). Overall, the behavior of 1D interacting gapless systems in the low-energy regime is well captured by the Tomonaga-Luttinger model (*2*–*4*) and has been extensively tested in carbon nanotubes (*5*–*7*), semiconductor quantum wires (*8*–*10*), antiferromagnets (*11*), and, more recently, cold-atom chains (*12*). The model, which assumes a linearized single-particle dispersion, is expected to only be valid close to the Fermi points, where nonlinearities are still weak. However, pronounced consequences of band curvature have also very recently started to be explored experimentally (*13*–*15*).

At the same time, modeling such systems is a long-standing open problem. Simultaneous introduction of the charge and spin degrees of freedom into a nonlinear extension of Tomonaga-Luttinger liquid (TLL) theory (*16*) predicted that the spin-charge separation would no longer exist beyond the low-energy regime (*17*, *18*), since the holons (i.e., charge-type excitations) are made unstable by the nonlinearities. Instead, a mixture of spinons (i.e., spin-type excitations) and holons is responsible for the power-law threshold behavior around the spectral edges. Extra, higher-order 1D modes, which have the spectral edge dispersion shifted and mirrored from that of the main 1D subband, were predicted in (*16*) as well. Another theory (*19*) showed that these extra modes should only emerge as the system length is reduced. We have observed some signatures of these “replicas” in the past (*20*, *21*), but experimental results beyond the linear regime that have both enough resolution and clarity to distinguish between predictions have been lacking.

Here, we measure the spectral function for the spin and charge excitations well beyond the linear regime using a tunneling spectroscopy technique that allows mapping in both energy and momentum space. In the amplitude of our signal, we find that the two branches of the linear TLL modes evolve away from the Fermi points into two fully formed nonlinear dispersions. Each dispersion consists of purely spin or charge collective modes, identified by comparison with the spectra predicted by the 1D Fermi-Hubbard model (*22*). Both dispersions are parabolic in shape but with different masses, implying the existence of two Fermi seas of different types. This result shows that the spin and charge collective excitations both remain stable in the whole conduction band, well beyond the low-energy limit of the original Tomonaga-Luttinger model where their existence was first established. We are able to tune the degree of screening of the Coulomb interaction by changing the confinement in our wires and hence the number of occupied subbands. This is accompanied by a variation of approximately 45% of the two-body interaction energy and allows us to trace how both Fermi seas evolve as the interaction strength is varied. Measuring wires of different lengths, we are also able to observe two, with a possible third, separate 1D nonlinear “replica” modes of the spinon type that systematically emerge as the length decreases.

## RESULTS

### Characterization of the 1D array

**Fig. 1.**
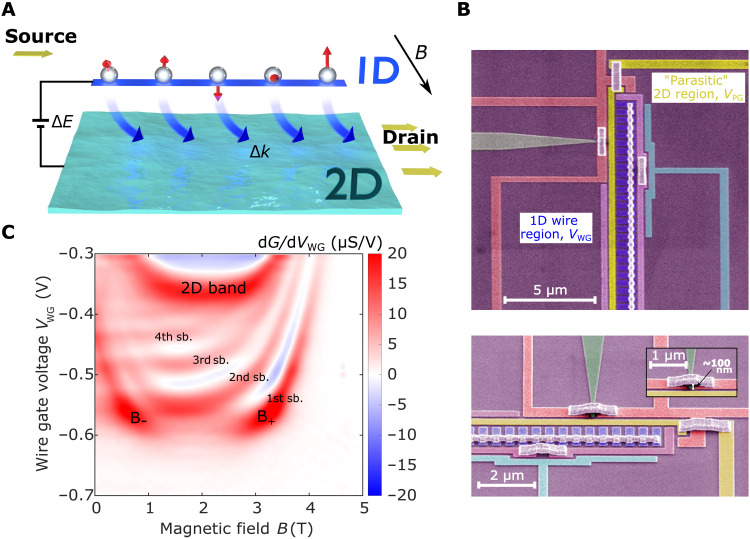
Mapping a 1D system via magnetotunneling spectroscopy. (**A**) Schematic representation of the 1D-2D spectrometer device. We measure momentum-resolved tunneling to and from an array of 1D wires (only one wire shown here for simplicity) and a 2D electron system and map the elementary excitations in each system by measuring the tunneling conductance while varying both their energy Δ*E* ∝ V_DC_ and momentum Δ*k* ∝ B. Current flows from the source into the wire and tunnels between the layers to reach the drain. (**B**) Scanning electron micrographs of the various surface gates present in our device. See Materials and Methods for details on gate operation and how to set up the tunneling regime. Inset: Air-bridge interconnections between surface gates. (**C**) 1D wire subbands (sb.) participating in the tunneling process. We observe from four to one 1D subbands before the wires pinch off.

Our experiment consists of a tunneling spectrometer made on a GaAs/Al_0.33_Ga_0.67_As heterostructure with two parallel quantum wells (QWs), grown by molecular beam epitaxy (MBE). We measure the tunneling conductance *G* = d*I*/d*V*_DC_ between the 1D wires and the 2D layer at lattice temperatures *T* ∼ 300 mK (see Fig. 1A), where *I* is the tunneling current, while *V*_DC_ is the DC bias applied between the layers. Tunneling occurs when filled states in one system have the same energy and momentum as empty states in the other, therefore ensuring that both energy and momentum are conserved. To map the dispersion of each system, a negative (positive) voltage *V*_DC_ applied to the 1D wires provides energy for tunneling from (to) 1D states below (above) the Fermi level, while an in-plane magnetic field *B* perpendicular to the wires boosts the momentum, offsetting the spectral functions of each system by Δ*k* = *eBd*/ħ, where *e* is the electronic charge, and *d* is the separation between the wells. The differential tunneling conductance *G* displays resonant peaks corresponding to maximal overlap of the offset spectral functions (dispersion relations). The device therefore behaves as a spectrometer, with the well-characterized 2D system being used to probe the less well-understood spectral function of the 1D system.

We use a surface-gate depletion technique to establish separate contacts to each well. Our 1D system consists of an array of ∼400 highly regular quantum wires formed in the upper layer by using a set of wire gates (WGs) fabricated on a Hall bar via standard electron beam lithography and connected by air bridges (see Fig. 1B and inset) (*23*). Use of an array averages out impurities, length resonances, and charging effects as well as increases the overall strength of the measured signal. For the shorter devices, the air bridges are crucial for ensuring that good uniformity is obtained along the entire length of the wire, which would otherwise become narrower at one end if instead all the gates were joined by a continuous metal strip. Current is injected into the 1D wires via a small region, 0.45 μm wide. Unlike the wires, however, this region is 2D in nature, and its parasitic signal can be readily distinguished from the 1D signal in the measured data since its density is different. We use the unconfined weakly interacting 2D electron gas (2DEG) in the bottom well as a well-understood spectrometer. Note that our measurement is subject to capacitive effects between the wells, as well as between each well and the surface gates, and we take these into account in all the curves we plot; see section S1.6.1.

**Fig. 2.**
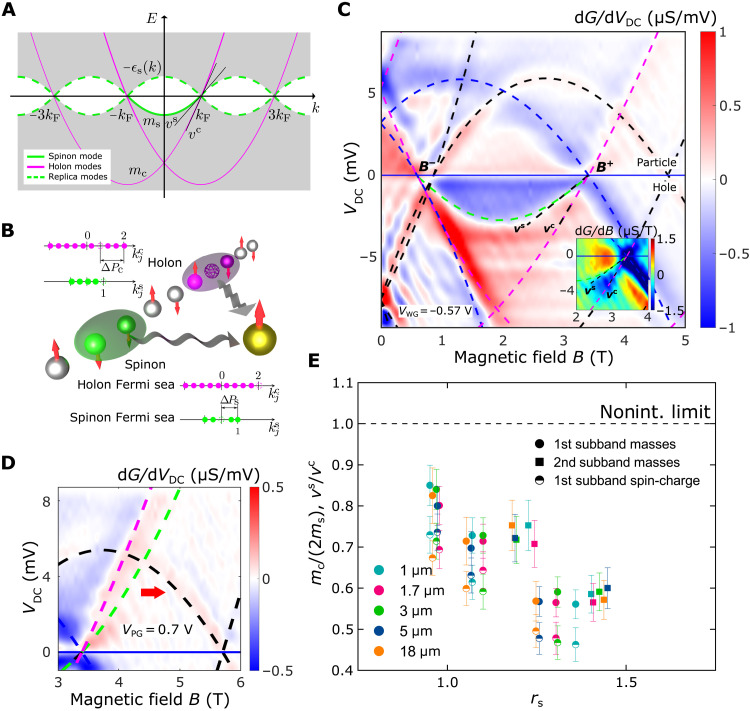
Two Fermi seas. (**A**) Dispersion proposed for an interacting 1D system (gray, continuum of many-body excitations; green lines, spinon modes; magenta lines, holon modes; dashed, replicas). (**B**) Graphical representation of the decomposition of an electron into a spinon and a holon in a tunneling process, along with an illustration of two distinct Fermi seas (filled dots) describing the pure holon (top) and spinon (bottom) excitations (in units of *k*_F_)—see text. (**C**) Map of the tunneling conductance (*G*) differential d*G*/d*V*_DC_ versus DC bias *V*_DC_ and in-plane magnetic field *B*, for a 5-μm-long device. Superimposed curves mark all possible single-electron tunneling processes, together with the resonant dispersions of the spin and charge modes marked by green and magenta dashed lines, respectively. Inset: d*G*/d*B* around the +*k*_F_ point at negative biases where both spin (*v*^s^) and charge (*v*^c^) lines can be seen. (**D**) d*G*/d*V*_DC_ above +*k*_F_(~3.3T), where *V*_PG_ = 0.7 V so that the parasitic signal has moved further to the right. We systematically observe no signal from the spinon mode at *V*_DC_ > 0 in all devices that we measured; see text for discussion. A naïve extension of the spinon dispersion, observed in the hole sector, into the particle sector is given by the green dashed line. (**E**) Ratio of holon-to-spinon masses and spinon-to-holon velocities versus interaction parameter *r*_s_ for devices of different lengths.

A plot of d*G*/d*V*_WG_ versus *B* and *V*_WG_ shows U-shaped curves, one per 1D subband (Fig. 1C). We start our experiment by choosing *V*_WG_ so that there is just one 1D subband occupied. Figure 2C shows an example of such a measurement, with conductance through the sample being measured as a function of energy (∝*V*_DC_) and momentum (∝*B*). Here, the tunneling map can be divided into two sectors, particle (for *V*_DC_ > 0) and hole (for *V*_DC_ < 0), corresponding, respectively, to electrons tunneling into and out of the wires. The 1D Fermi wave vector *k*_1D_ = *ed*(*B*^+^ − *B*^−^)/2ħ is determined from the crossing points (*B*^−^ and *B*^+^) along the *V*_DC_ = 0 line. The electron density in the wires is *n*_1D_ = 2*k*_1D_/π, which then gives the interaction parameter $r_{s}=1/(2a_{B}^{\prime }n_{1D})$, where $a_{B}^{\prime }$ is the Bohr radius of conduction electrons in GaAs; see section S1.5. The density can be controlled by tuning *V*_WG_, reducing it down to *n*_1D_ ∼ 30 μm^−1^ before the wires pinch off.

### Observation of two Fermi seas for spin and charge

The curves drawn over the data in Fig. 2C are those expected from single-electron tunneling processes. Undesirable yet unavoidable “parasitic” tunneling coming from the narrow 2D injection region (marked by the black dashed curves; see section S1.6.2) produces a background in the form of a set of parabolic dispersions, which can be subtracted once separately mapped (with the WGs pinched off). On the other hand, blue dashed curves reveal the elementary excitations of the 2D lower well, as probed by the 1D wires. The remaining strong features, marked by the green and magenta dashed curves, arise from the 1D system. We are unable to explain these data well using a single parabola (see section S1.6.3 for details), a fact that strongly points in the direction of separate spin and charge modes.

To identify whether this is the case, we interpret the 1D tunneling signal using the dispersion of the 1D Fermi-Hubbard model in the semiconductor limit, in which many-body spectra are described completely by the Lieb-Wu equations (*22*). This system of nonlinear coupled equations is solved for two types of momentum states, $k_{j}^{c}$ (for charge) and $k_{j}^{s}$ (for spin degrees of freedom), which, for the ground state, form two filled Fermi seas marked by filled circles of two different colors in Fig. 2B (for a detailed discussion of the theoretical model, see sections S1.1, S1.2, and S1.3). An excitation, say an electron tunneling out of the wire, removes one charge and one spin simultaneously to reassemble a free electron, marked by a pair of green and magenta empty circles in Fig. 2B. Placing the hole of one type at its corresponding Fermi energy and moving the other one through the band describes the spectrum of the purely holon or purely spinon modes. While the momentum of these collective excitations as a whole is well defined because of the translational invariance, with *k* = *k*_F_ − Δ*P*_c_ or *k* = *k*_F_ − Δ*P*_s_, the constituent degrees of freedom form nonequidistant distributions of their (quasi-)momenta, which depend, in detail, on the interaction strength and the positions of the two holes, owing to the strongly correlated nature of the model. Explicit solution of the Lieb-Wu equations for $k_{j}^{c}$ and $k_{j}^{s}$ for the two kinds of pure excitations produces the two dispersions drawn as the magenta and green solid lines in Fig. 2A. These two dispersions constructed out of collective modes for spin and charge have a shape close to parabolic and have a simple description in terms of two Fermi seas with different masses. This closely matches our experimental observations, implying the presence of these two Fermi seas (see section S1.6) in a real system of interacting electrons.

Around the ±*k*_F_ points, these curves are almost linear and can be characterized by two different slopes *v*^c^ and *v*^s^, parameters of the spinful Tomonaga-Luttinger model for the holon and spinon modes, respectively. These two velocities are related microscopically to the Hubbard interaction parameter *U* (*24*, *25*), and the spectral function predicted by the linear Tomonaga-Luttinger theory displays two strong peaks on these two branches (*26*, *27*), which have already been measured in semiconductor quantum wires (*8*, *9*). Away from the Fermi points, the spectra of holons and spinons extend naturally to the nonlinear region, evolving into two separate curves that are close to parabolae described by masses *m*_c_ and *m*_s_, respectively. These shapes indicate the formation of two separate Fermi seas by the nonlinear excitations. Their dispersions cross the Fermi energy at two different pairs of Fermi points (±*k*_F_ and ±3*k*_F_) [see a numerical simulation of the Fermi-Hubbard model via the dynamical density-matrix renormalization group method in (*28*)], since the number of holons is twice the number of spinons for the spin-unpolarized wires in our experiments, making the densities for the two kinds also different by the same factor. The ratio of their masses depends on *U*, deviating further from the free-particle value *m*_c_/(2*m*_s_) = 1 with increasing interaction strength.

As we have seen, the dispersion of the strongest features in the experimental 1D signal (marked by green and magenta dashed lines in Fig. 2C) cannot simply be interpreted using only a single parabola, corresponding to a single Fermi sea. This can be further established by analyzing the tunneling signal at zero field; see section S1.6.4 for details. If, instead of just one, we use two parabolae, corresponding to two Fermi seas, such as that predicted by the Fermi-Hubbard model, then we can match the experiments well. We observe two modes in our data, which match the dispersions of pure excitations of the two different kinds depicted in Fig. 2A, where two distinct Fermi seas are formed by the nonlinear spinon and holon collective modes out of the many-body continuum away from the Fermi points. This result demonstrates a nonperturbative effect that interactions cause in the whole band in 1D, posing a new theoretical challenge of accounting for higher-order processes beyond what has been considered in the literature so far (*29*) to describe it.

While we observe two dispersions in the hole sector, only one is visible in the particle sector, which we easily associate with the holon Fermi sea, as its gradient matches that of the charge line in the hole sector. At the same time, the spinon dispersion that assumes a naïve extension from the hole sector is systematically absent in the particle sector for all devices measured; see green dashed line in Fig. 2D. This result is, however, compatible with the particle-hole asymmetry in relaxation times of hot carriers as reported previously in (*13*). Although spin and charge excitations were not resolved in (*13*), assuming that the spinon branch in the particle sector is unstable (so that we do not observe it), there would be an accelerated relaxation for hot electrons as they eventually split into spinon and holons. Note that the spinon Fermi sea alone has already been observed by neutron scattering (probing the dynamic structure factor instead of the spectral function that we measure here) in antiferromagnetic spin chains realized in insulating materials (*30*–*32*) as a spectral edge with a nonlinear dispersion separating the multispinon continuum from a forbidden region (*33*, *34*). In these experiments, the spectral power of the excitations drops very rapidly toward the particle part of the spinon dispersion, making it therefore undetectable. Also note that, in the present experiment, the charges are delocalized as well, permitting us to see both Fermi seas at the same time.

By tuning the confinement in the wires, we are also able to change the number of occupied subbands and their respective densities, therefore allowing us to follow the evolution of each Fermi sea as *r*_s_ is changed by a substantial amount. Such statistics collected from a range of samples in Fig. 2E show a systematic trend of larger deviations of the observed *m*_c_/(2*m*_s_) ratio from its noninteracting value with increasing *r*_s_. The ratio of the Luttinger parameters *v*^s^/*v*^c^ simultaneously extracted from the same data (see as an example the inset in Fig. 2C) exhibits a very similar dependence on *r*_s_.

### Further interaction signatures: A hierarchy of 1D replica modes

**Fig. 3.**
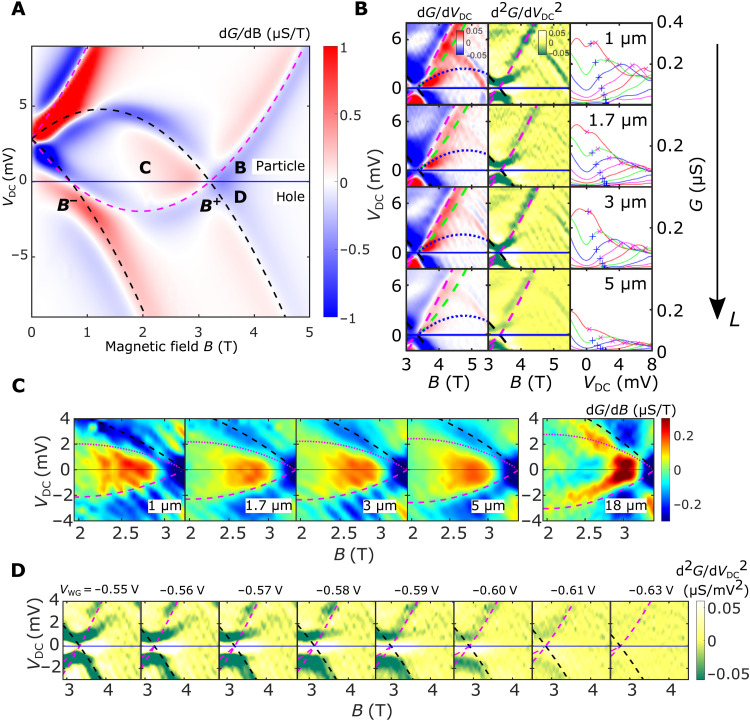
A hierarchy of modes. (**A**) Simulated map of the differential tunneling conductance d*G*/d*B* versus *V*_DC_ and *B*, between a 1D noninteracting system (magenta) and a 2DEG (black). In the absence of interactions, the spinon and holon dispersions are degenerate with each other. (**B**) d*G*/d*V*_DC_ (left) and $d^{2}G/dV_{\text{DC}}^{2}$ (center), for devices of different lengths, as labeled. Right column: *G* versus *V*_DC_ at various fields *B* > *B*^+^ for the data in the matching plots to the left; “×” and “+” symbols on each curve indicate the position of the fitted dispersions in the particle sector for the holon branch and the first-order replica, respectively (see text for definition); *G* stays high between the two. (**C**) d*G*/d*B* for *B* < *B*^+^, showing another first-order replica mode (magenta dotted line) in the particle sector for a variety of different-length devices. (**D**) $d^{2}G/dV_{\text{DC}}^{2}$ for a 1-μm device at a variety of different WG voltages *V*_WG_. This replica mode responds to changes in *V*_WG_, completely disappearing once the wires are pinched off. Symmetric to it, in the hole sector, a kink in conductance can be observed, only visible in our shortest 1-μm devices. Conductance has been normalized by device length in (B) to (D).

Having now identified two separate Fermi seas for spin and charge in our data, we further analyze the 1D dispersion by contrasting it with the simulated tunneling conductance map between a noninteracting 1D system and a 2DEG (see Fig. 3A). Note how, unlike in Fig. 2C, it is possible to fit both particle and hole sectors of the map with a single parabola (magenta dashed line). This is because, in the absence of interactions, the opposite spin states are degenerate, leaving room for only a single Fermi sea. We start by examining the region just above *B*^+^ (i.e., +*k*_F_), where a clear feature not accounted for by our noninteracting simulation can be observed; see Fig. 3B. Here, the tunneling conductance peak broadens and splits, with one boundary following the 1D holon mode while the other branches away from it. To isolate it from any potential background contamination, we apply a positive *V*_PG_ again to move the parasitic resonance signatures away from *B*^+^. We also observe that this extra feature is not visible once the wires are past pinch-off and that it is independent of the parasitic tunneling signal.

The mode-hierarchy picture for fermions (*19*, *20*) predicts that the continuum of the many-body excitations separates itself into levels (i.e., first and higher orders) by their spectral strengths, which are proportional to integer powers of *R*^2^/*L*^2^ (i.e., this parameter to the first and higher powers), where *R* is the length scale related to the interaction, and *L* is the length of the system (see section S1.4 for more details). The principal (spin and charge) parabolae have then the largest amplitude, while their mirrors with respect to the chemical potential (i.e., *V*_DC_ = 0) and translations by integer multiples of 2*k*_F_ manifest themselves as replicas; see dashed lines on Fig. 2A, with amplitudes proportional to higher powers of the small parameter *R*^2^/*L*^2^. We have observed this feature in samples with wire lengths ranging from 1 to 18 μm, with all devices being mapped at very similar densities and Fermi energies, making them otherwise similar in *R*. In all of them, the strength of the mode marked by the blue dotted line in Fig. 3B, which is a replica of the parabola formed by nonlinear spinons, decreases as the *B* field is increased away from the crossing point. However, once the background has been subtracted and *G* has been normalized by length, one can see qualitatively that the decay away from *B*_+_ was slower the shorter the 1D system, as predicted, with the signal vanishing at higher momenta away from +*k*_F_.

To test our prediction of length-dependent spin-type replica modes further, we have looked at two other sectors of the tunneling map; see Fig. 3 (C and D). We initially reported the first mode between ±*k*_F_ as an inverted (spinon) shadow band symmetric to the 1D (spinon) mode in (*21*). According to the nonlinear theory of TLLs (*16*, *35*), in the main ∣*k* ∣ < *k*_F_ region of the hole sector, the edge of support (defined as the hole excitation with the smallest possible energy for a given momentum) is predicted (*17*, *36*) to coincide with the spinon mass shell, whose dispersion ϵ_s_(*k*) we have already observed to be very close to a parabola in our experiment. Similarly, in the main region of the particle sector, the edge of support is also predicted to be given by the inverted spinon mass shell −ϵ_s_(*k*) in Fig. 2A. Consistent with the nonlinear theory, a symmetric inverted replica was seen in the particle sector, opposite to the main 1D subband, in all mapped devices, up to 5 μm (see Fig. 3C). This feature can also be seen in Fig. 2C. According to the mode-hierarchy picture, a length dependence similar to that of Fig. 3B is also expected to be observed since this is also a subleading mode. Although such dependence is not particularly clear from 1 to 5 μm, the replica mode was seen to not be present at all for the 18-μm wire.

Similarly, another replica mode is also predicted to exist at *k*_F_ < *k* < 3*k*_F_, symmetric to the subleading spinon mode shown in Fig. 3B, but in the hole sector. For only the shortest 1-μm device, a feature consistent with this picture is starting to be observed, hinting that a full observation of this mode would probably only happen at submicron lengths. Nevertheless, as seen in Fig. 3D, both modes evolve in tandem with each other as the 1D channels are squeezed toward pinch-off. This further establishes that these features are 1D in nature and cannot originate from the parasitic injection region. All three replica features discussed emerge as the effective length of the 1D system is reduced, compatible with the mode-hierarchy picture where a level hierarchy emerges controlled by the system’s length. We attribute the different lengths at which they become visible in this experiment (the first replica only being observed below 5 μm, the second below 18 μm, and the third replica only at 1 μm) to different numerical prefactors that are still unknown theoretically for spinful systems. Nevertheless, the fact that we are observing features compatible with the mode-hierarchy picture further establishes our technique as being capable of detecting interaction effects in the nonlinear regime.

### Evolution of the two Fermi seas with interaction strength

**Fig. 4.**
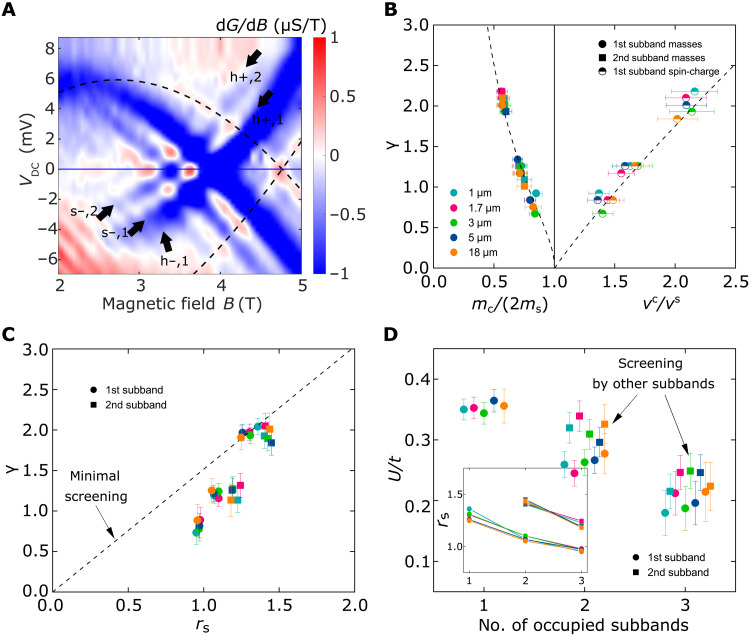
1D-1D screening. (**A**) d*G*/d*B* of a 5-μm device mapped in the multisubband regime. Black dashed line marks the location of the subtracted 2D-2D parasitic signal. Arrows point to the location of spinon (s) and holon (h) modes, in both the hole (−) and particle (+) sectors, for each occupied subband (1 and 2). (**B**) Macroscopic dimensionless Hubbard-model parameter γ (see text for discussion) versus mass and velocity ratio, for devices of different lengths, as extracted by fitting the bottom two subbands. The Hubbard model (black dashed lines) can reproduce well the observed experimental dependence. (**C**) γ versus the interaction parameter *r*_s_, where an approximate linear dependence can be seen. The dashed curve corresponds to fitting using only data from the single-subband-occupancy regime. Note that, even when allowing for errors, all remaining points fall systematically below this line, indicating the presence of 1D-1D intersubband screening. (**D**) Hubbard parameter *U*/*t* versus the number of occupied subbands as extracted from γ. The asymmetry in screening between the first and second subbands is expected from their difference in densities. Points slightly offset horizontally from each other for clarity. Inset: Interaction parameter *r*_s_ versus the number of occupied subbands. We can change *r*_s_ by tuning the 1D density *n*_1D_ of the wires (see fig. S4B in the Supplementary Materials).

Up to now, we have confined our analysis to dispersion maps in the single-subband regime. In the current geometry, however, we are also able to vary the number of occupied subbands up to four, by tuning the WG voltage *V*_WG_ until the upper layer starts to become 2D when carriers delocalize between the wires. While the emergent hierarchy of modes becomes almost impossible to see in the data with more than one subband occupied, the parameters of two Fermi seas can still be quite reliably extracted; see Fig. 4A and section S1.6.5. Variation of the number of subbands provides us with an additional tool for assessing the microscopic interaction parameter of the Hubbard parameter *U* in our experiment at the quantitative level. The macroscopic dimensionless parameter controlling the Hubbard model in 1D is (*37*)

$$\gamma =0.032\frac{\lambda_{F}}{a}\frac{U}{t}$$ (1)

where *a* is the lattice parameter of the underlying crystal, *t* is the hopping amplitude, and λ_F_ is the Fermi wavelength. For γ < 1, the weakly interacting electrons are almost spin-degenerate, having double occupancy for each momentum state, as for free particles. For γ > 1, each momentum state is occupied by only one electron due to strong Coulomb repulsion. Such a dependence of the system’s behavior on γ is qualitatively the same as the dependence on *r*_s_ in all dimensions, reflecting the ratio of the total interaction energy to the kinetic energy.

We have varied the number of occupied subbands all the way up to four and fitted the bottom two using the model of two Fermi seas to extract the ratio of masses and velocities. Fitting the same data with the dispersion produced by the Hubbard model in a similar fashion to what was done before for the single-subband regime, see Fig. 2C, we obtain the values of γ that correspond to these ratios for each individual subband and for each subband occupancy in Fig. 4B, which allows the data points from multiple wires with a variety of densities (as seen in Fig. 2E) to be collapsed onto the same curve. The agreement between experiment and theory seen here further reinforces our claim of two separate Fermi seas for spinons and holons, having collected statistics belonging to five different devices, fabricated from two different wafers, across different fabrication cycles, and measured independently of one another in different cooldowns. Comparing the already-extracted values of *r*_s_ with γ for all measurements in Fig. 4C, we find that the two are approximately proportional to each other with a coefficient of ≃1.5. We interpret the still-observable discrepancy as a manifestation of the screening effect that is not captured by *r*_s_ but is taken into account explicitly in the Hubbard model via the two-body interaction energy *U*. The latter is proportional to the integral of the screened Coulomb potential, in which only the screening radius is changed in our experiment.

By means of the relation in Eq. 1, we extract the evolution of *U*/*t* from the already-obtained values of γ and λ_F_ as a function of the number of occupied subbands, as shown in Fig. 4D, for both the first and second lowest subbands, in different-length systems. Data corresponding to four occupied subbands were excluded, as its proximity to the noninteracting limit made the fitting less reliable. Similarly, fitting to the dispersions of the third and fourth subbands was not attempted owing to the lack of sharp features and overall increase in map complexity. Nevertheless, two clear trends emerge: First, *U* decreases as more subbands are progressively filled, resulting in relative reductions of about ∼45% for the first subband and of ∼25% for the second subband; second, the bottom subband seems to be systematically more strongly screened than the second, most likely due to their difference in density. These trends are also apparent in Fig. 4C, where the dashed line is drawn through the single-subband points, and all further occupied subbands systematically fall below it. We note that the values measured in the single-subband regime are consistent with the estimates made in (*38*), where the long-range Coulomb interaction between the electrons is screened by a conducting plate a certain distance away from the 1D wire. As far as the authors are aware, this is the first clear observation of screening effects between two 1D systems, with similar conclusions reported in (*39*) in 2D systems.

## DISCUSSION

We have shown that spin-charge separation is more robust than previously thought, extending past the low-energy regime of the TLL to beyond the Fermi energy. By tuning the degree of screening of the Coulomb interaction by changing the confinement in our wires and hence the number of occupied subbands, we saw how the masses associated with the spin and charge Fermi seas evolve as a function of the interaction strength, with a remarkably good quantitative agreement with the predictions of the 1D Fermi-Hubbard model. At the same time, our comparison of quantum wires of different lengths confirms the prediction of the mode-hierarchy theory, observing systematically the emergence of at least two replica modes as the wire length decreases.

## MATERIALS AND METHODS

### Device fabrication

All tunneling devices measured in this work were fabricated using double-QW heterostructures, grown via MBE, composed of two identical 18-nm GaAs QWs separated by a 14-nm Al_0.165_Ga_0.835_As superlattice tunnel barrier (10 pairs of Al_0.33_Ga_0.67_As and GaAs monolayers). On each side of the barrier, there were 40-nm Si-doped layers of Al_0.33_Ga_0.67_As (donor concentration, 1 × 10^24^ m^−3^), with the lower and upper spacers being 40 and 20 nm wide, respectively. Wafer 1, however, differed from wafer 2 by having an additional 100 ×2.5 mm/2.5 mm GaAs/AlGaAs superlattice below the 350-nm AlGaAs under the lower QW. This resulted in electron concentrations of about 3 (2.2) ×10^15^ m^−2^ with mobilities of around 120 (165) m^2^ V^−1^ s^−1^ in the top (bottom) well of wafer 1 while 2.85 (1.54) ×10^15^ m^−2^ and 191 (55) m^2^ V^−1^ s^−1^ for wafer 2, as measured at 1.4 K. A 10-nm GaAs cap layer was used to prevent oxidation. The distance from the upper well to the surface was ∼70 nm.

**Fig. 5.**
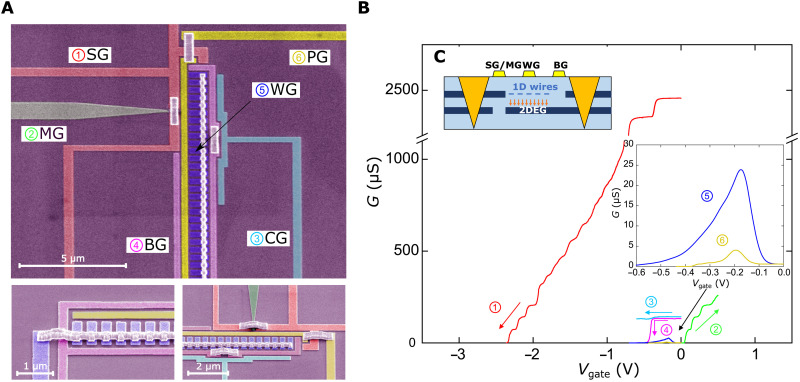
Vertical tunneling device. (**A**) Scanning electron microscopy images of the tunneling device, showing the SG, MG, BG, WG, and PG. The CG is not used in this work and is always biased together with BG. Several samples were fabricated to vary the length of WG from 1 to 18 μm (pictured, 1 μm). The bottom micrographs show, respectively, the lower and upper ends of the wire array (∼400 wires). To increase the uniformity of the 1D system, we developed a novel air-bridge technique to avoid having to use a connecting backbone structure [see (*23*)]. (**B**) Gate operation and setting of tunneling conditions. We start by negatively biasing SG (1), followed by positively biasing MG so that conductance is allowed only in the upper well (UW) (2). Next, we negatively bias both CG (3) and BG (4) but enough to only deplete the UW. Under this configuration, any signal measured between the ohmic contacts must result from direct tunneling between each well. Inset: By varying WG and/or PG, one can observe, respectively, 1D-2D and 2D-2D tunneling between the wells (5 and 6). (**C**) Side profile of the tunneling device. It consists of a double QW heterostructure with a center-to-center distance of about ∼32 nm.

The electrical (surface) structure of the device was fabricated on a 200-μm-wide Hall bar. Contacts to both layers were established using AuGeNi ohmic contacts. Electron beam lithography was used to define a split gate (SG), a midline gate (MG), a barrier gate (BG), and a cutoff gate (CG)—used in setting up the tunneling conditions—together with an array of WGs—used in defining the experimental 1D system (see Fig. 5A). The length of the WGs was varied from 1 to 18 μm. They were separated by 0.15- to 0.18-μm gaps and had a width of 0.1 to 0.3 μm. A parasitic injection region also ran across the entire width of the mesa, with a fixed width of 0.45 μm. A parasitic gate (PG) was used to modulate its density. All dimensions, particularly regarding the wire region, were carefully chosen to achieve minimal modulation of the lower-well carriers.

### Momentum- and energy-resolved tunneling spectroscopy

The tunneling setup was achieved as follows: First, the SG was negatively biased to pinch off both layers underneath, followed by positively biasing the MG to open a narrow conducting channel in the top well. At the other end of the device, the BG and the CG were biased enough to pinch off only the top layer. Under these conditions, any current injected through one of the ohmic contacts had to have tunneled between the layers to be detected (Fig. 5B).

Our spectroscopy technique consists of a low-noise, low-temperature measurement of the tunneling current between the two 2DEG layers, which is given by (*29*)

$$\begin{array}{c} I\propto \int dkdE[f_{T}(E-E_{F1D}-{\mathrm{eV}}_{\text{DC}})-f_{T}(E-E_{F2D})]\\ \times A_{1}(k,E)A_{2}(k+\mathrm{ed}(n\times B)/\hbar ,E-{\mathrm{eV}}_{\text{DC}}) \end{array}$$ (2)

where *e* is the electron charge, *f_T_*(*E*) is the Fermi-Dirac distribution function, *d* is the center-to-center well separation, **n** is the unit normal to the 2D plane, $B=-B\hat{y}$ is the magnetic field vector, $\hat{y}$ is the unit vector in the *y* direction, and *A*_1_(**k**, *E*) and *A*_2_(**k**, *E*) are the spectral functions of the 1D and 2D systems, respectively, with the corresponding Fermi energies being *E*_F1D_ and *E*_F2D_. The tunneling current between the two layers is then proportional to the overlap integral of their spectral functions. We induce an offset *eV*_DC_ between the Fermi energies of the two systems by applying a DC bias *V*_DC_ between the layers. Similarly, an offset in momentum can also be obtained by applying a magnetic field of strength *B* parallel to the 2DEG layers. When the field direction is along the (in-plane) *y* direction, the Lorentz force then shifts the momentum of the tunneling electrons in the *x* direction by *edB*. Put together, one can therefore map the dispersion of each system with respect to one another by measuring the differential conductance *G* = d*I*/d*V* in both energy and momentum space.

## Supplementary Materials

### This PDF file includes:

Supplementary Text Figs. S1 to S9 References

## References

[1] T. Giamarchi, *Quantum Physics in One Dimension* (Clarendon Press, 2003).

[2] S. Tomonaga, Remarks on Bloch’s method of sound waves applied to many-fermion problems. Prog. Theor. Phys. 5, 544–569 (1950).

[3] J. M. Luttinger, An exactly soluble model of a many-fermion system. J. Math. Phys. 4, 1154–1162 (1963).

[4] F. D. M. Haldane, ‘Luttinger liquid theory’ of one-dimensional quantum fluids. I. Properties of the Luttinger model and their extension to the general 1D interacting spinless Fermi gas. J. Phys. C Solid State Phys. 14, 2585–2609 (1981).

[5] M. Bockrath, D. H. Cobden, J. Lu, A. G. Rinzler, R. E. Smalley, L. Balents, P. L. McEuen, Luttinger-liquid behaviour in carbon nanotubes. Nature 397, 598–601 (1999).

[6] H. Ishii, H. Kataura, H. Shiozawa, H. Yoshioka, H. Otsubo, Y. Takayama, T. Miyahara, S. Suzuki, Y. Achiba, M. Nakatake, T. Narimura, M. Higashiguchi, K. Shimada, H. Namatame, M. Taniguchi, Direct observation of Tomonaga-Luttinger-liquid state in carbon nanotubes at low temperatures. Nature 426, 540–544 (2003). 14654836 10.1038/nature02074

[7] Z. Shi, X. Hong, H. A. Bechtel, B. Zeng, M. C. Martin, K. Watanabe, T. Taniguchi, Y.-R. Shen, F. Wang, Observation of a Luttinger-liquid plasmon in metallic single-walled carbon nanotubes. Nat. Photonics 9, 515–519 (2015).

[8] O. M. Auslaender, H. Steinberg, A. Yacoby, Y. Tserkovnyak, B. I. Halperin, K. W. Baldwin, L. N. Pfeiffer, K. W. West, Spin-charge separation and localization in one dimension. Science 308, 88–92 (2005). 15802599 10.1126/science.1107821

[9] Y. Jompol, C. J. B. Ford, J. P. Griffiths, I. Farrer, G. A. C. Jones, D. Anderson, D. A. Ritchie, T. W. Silk, A. J. Schofield, Probing spin-charge separation in a Tomonaga-Luttinger liquid. Science 325, 597–601 (2009). 19644117 10.1126/science.1171769

[10] D. Laroche, G. Gervais, M. P. Lilly, J. L. Reno, Positive and negative Coulomb drag in vertically integrated one-dimensional quantum wires. Nat. Nanotechnol. 6, 793–797 (2011). 22036809 10.1038/nnano.2011.182

[11] B. J. Kim, H. Koh, E. Rotenberg, S.-J. Oh, H. Eisaki, N. Motoyama, S. Uchida, T. Tohyama, S. Maekawa, Z.-X. Shen, C. Kim, Distinct spinon and holon dispersions in photoemission spectral functions from one-dimensional SrCuO_2_. Nat. Phys. 2, 397–401 (2006).

[12] J. Vijayan, P. Sompet, G. Salomon, J. Koepsell, S. Hirthe, A. Bohrdt, F. Grusdt, I. Bloch, C. Gross, Time-resolved observation of spin-charge deconfinement in fermionic Hubbard chains. Science 367, 186–189 (2020). 31919220 10.1126/science.aay2354

[13] G. Barak, H. Steinberg, L. N. Pfeiffer, K. W. West, L. Glazman, F. von Oppen, A. Yacoby, Interacting electrons in one dimension beyond the Luttinger-liquid limit. Nat. Phys. 6, 489–493 (2010).

[14] Y. Jin, O. Tsyplyatyev, M. Moreno, A. Anthore, W. K. Tan, J. P. Griffiths, I. Farrer, D. A. Ritchie, L. I. Glazman, A. J. Schofield, C. J. B. Ford, Momentum-dependent power law measured in an interacting quantum wire beyond the Luttinger limit. Nat. Commun. 10, 2821 (2019). 31249302 10.1038/s41467-019-10613-2 PMC6597579

[15] S. Wang, S. Zhao, Z. Shi, F. Wu, Z. Zhao, L. Jiang, K. Watanabe, T. Taniguchi, A. Zettl, C. Zhou, F. Wang, Nonlinear Luttinger liquid plasmons in semiconducting single-walled carbon nanotubes. Nat. Mater. 19, 986–991 (2020). 32231241 10.1038/s41563-020-0652-5

[16] A. Imambekov, L. I. Glazman, Universal theory of nonlinear Luttinger liquids. Science 323, 228–231 (2009). 19039106 10.1126/science.1165403

[17] T. L. Schmidt, A. Imambekov, L. I. Glazman, Fate of 1D spin-charge separation away from Fermi points. Phys. Rev. Lett. 104, 116403 (2010). 20366492 10.1103/PhysRevLett.104.116403

[18] T. L. Schmidt, A. Imambekov, L. I. Glazman, Spin-charge separation in one-dimensional fermion systems beyond luttinger liquid theory. Phys. Rev. B 82, 245104 (2010).

[19] O. Tsyplyatyev, A. J. Schofield, Y. Jin, M. Moreno, W. K. Tan, C. J. B. Ford, J. P. Griffiths, I. Farrer, G. A. C. Jones, D. A. Ritchie, Hierarchy of modes in an interacting one-dimensional system. Phys. Rev. Lett. 114, 196401 (2015). 26024184 10.1103/PhysRevLett.114.196401

[20] O. Tsyplyatyev, A. J. Schofield, Y. Jin, M. Moreno, W. K. Tan, A. S. Anirban, C. J. B. Ford, J. P. Griffiths, I. Farrer, G. A. C. Jones, D. A. Ritchie, Nature of the many-body excitations in a quantum wire: Theory and experiment. Phys. Rev. B 93, 075147 (2016).

[21] M. Moreno, C. J. B. Ford, Y. Jin, J. P. Griffiths, I. Farrer, G. A. C. Jones, D. A. Ritchie, O. Tsyplyatyev, A. J. Schofield, Nonlinear spectra of spinons and holons in short GaAs quantum wires. Nat. Commun. 7, 12784 (2016). 27627993 10.1038/ncomms12784 PMC5027612

[22] E. H. Lieb, F. Y. Wu, Absence of Mott transition in an exact solution of the short-range, one-band model in one dimension. Phys. Rev. Lett. 20, 1445–1448 (1968).

[23] Y. Jin, M. Moreno, P. M. T. Vianez, W. K. Tan, J. P. Griffiths, I. Farrer, D. A. Ritchie, C. J. B. Ford, Microscopic metallic air-bridge arrays for connecting quantum devices. Appl. Phys. Lett. 118, 162108 (2021).

[24] H. J. Schulz, Correlation exponents and the metal-insulator transition in the one-dimensional hubbard model. Phys. Rev. Lett. 64, 2831–2834 (1990). 10041822 10.1103/PhysRevLett.64.2831

[25] H. Frahm, V. E. Korepin, Critical exponents for the one-dimensional Hubbard model. Phys. Rev. B 42, 10553–10565 (1990). 10.1103/physrevb.42.10553 9995315

[26] V. Meden, K. Schönhammer, Spectral functions for the Tomonaga-Luttinger model. Phys. Rev. B 46, 15753–15760 (1992). 10.1103/physrevb.46.15753 10003715

[27] J. Voit, Charge-spin separation and the spectral properties of Luttinger liquids. Phys. Rev. B 47, 6740–6743 (1993). 10.1103/physrevb.47.6740 10004648

[28] H. Benthien, F. Gebhard, E. Jeckelmann, Spectral function of the one-dimensional hubbard model away from half filling. Phys. Rev. Lett. 92, 256401 (2004). 15245039 10.1103/PhysRevLett.92.256401

[29] G. D. Mahan, *Many-Particle Physics* (Plenum, 1990).

[30] B. Lake, D. A. Tennant, C. D. Frost, S. E. Nagler, Quantum criticality and universal scaling of a quantum antiferromagnet. Nat. Mater. 4, 329–334 (2005). 15778717 10.1038/nmat1327

[31] M. Mourigal, M. Enderle, A. Klöpperpieper, J.-S. Caux, A. Stunault, H. M. Rønnow, Fractional spinon excitations in the quantum Heisenberg antiferromagnetic chain. Nat. Phys. 9, 435–441 (2013).

[32] B. Lake, D. A. Tennant, J.-S. Caux, T. Barthel, U. Schollwöck, S. E. Nagler, C. D. Frost, Multispinon continua at zero and finite temperature in a near-ideal Heisenberg chain. Phys. Rev. Lett. 111, 137205 (2013). 24116814 10.1103/PhysRevLett.111.137205

[33] J.-S. Caux, J. M. Maillet, Computation of dynamical correlation functions of Heisenberg chains in a magnetic field. Phys. Rev. Lett. 95, 077201 (2005). 16196817 10.1103/PhysRevLett.95.077201

[34] F. Göhmann, A. Klümper, A. Seel, Integral representations for correlation functions of the XXZ chain at finite temperature. J. Phys. A 37, 7625–7651 (2004).

[35] A. Imambekov, T. L. Schmidt, L. I. Glazman, One-dimensional quantum liquids: Beyond the Luttinger liquid paradigm. Rev. Mod. Phys. 84, 1253–1306 (2012).

[36] F. H. L. Essler, Threshold singularities in the one-dimensional Hubbard model. Phys. Rev. B 81, 205120 (2010).

[37] O. Tsyplyatyev, A. J. Schofield, Spectral-edge mode in interacting one-dimensional systems. Phys. Rev. B 90, 014309 (2014).

[38] L. I. Glazman, I. M. Ruzin, B. I. Shklovskii, Quantum transport and pinning of a one-dimensional Wigner crystal. Phys. Rev. B 45, 8454–8463 (1992). 10.1103/physrevb.45.8454 10000682

[39] M. Kim, S. G. Xu, A. I. Berdyugin, A. Principi, S. Slizovskiy, N. Xin, P. Kumaravadivel, W. Kuang, M. Hamer, R. K. Kumar, R. V. Gorbachev, K. Watanabe, T. Taniguchi, I. V. Grigorieva, V. I. Fal’ko, M. Polini, A. K. Geim, Control of electron-electron interaction in graphene by proximity screening. Nat. Commun. 11, 2339 (2020). 32393747 10.1038/s41467-020-15829-1 PMC7214472

[40] M. Gaudin, Un systeme a une dimension de fermions en interaction. Phys. Lett. A 24, 55–56 (1967).

[41] C. N. Yang, Some exact results for the many-body problem in one dimension with repulsive delta-function interaction. Phys. Rev. Lett. 19, 1312–1315 (1967).

[42] R. Orbach, Linear antiferromagnetic chain with anisotropic coupling. Phys. Rev. 112, 309–316 (1958).

[43] M. Ogata, H. Shiba, Bethe-ansatz wave function, momentum distribution, and spin correlation in the one-dimensional strongly correlated Hubbard model. Phys. Rev. B 41, 2326–2338 (1990). 10.1103/physrevb.41.2326 9993968

[44] M. Gaudin, *The Bethe Wavefunction* (Cambridge Press, 2014).

[45] C. F. Coll III, Excitation spectrum of the one-dimensional Hubbard model. Phys. Rev. B 9, 2150–2158 (1974).

[46] P. Vianez, O. Tsyplyatyev, C. Ford, Chapter Three - Semiconductor nanodevices as a probe of strong electron correlations, in *Frontiers of Nanoscience*, D. A. Ritchie, Ed. (Elsevier, 2021), vol. 20 of *Semiconductor Nanodevices*, pp. 31–66.

[47] S. E. Laux, D. J. Frank, F. Stern, Quasi-one-dimensional electron states in a split-gate GaAs/AlGaAs heterostructure. Surf. Sci. 196, 101–106 (1988).

[48] E. Owen, C. Barnes, Ground-state electronic structure of quasi-one-dimensional wires in semiconductor heterostructures. Phys. Rev. Appl. 6, 054007 (2016).

[49] S. Luryi, Quantum capacitance devices. Appl. Phys. Lett. 52, 501–503 (1988).

[50] P. T. Coleridge, M. Hayne, P. Zawadzki, A. S. Sachrajda, Effective masses in high-mobility 2D electron gas structures. Surf. Sci. 361-362, 560–563 (1996).

[51] M. Hayne, A. Usher, J. J. Harris, C. T. Foxon, Exchange enhancement of the Landau-level separation for two-dimensional electrons in GaAs/Ga_1 − *x*_Al*_x_*As heterojunctions. Phys. Rev. B 46, 9515–9519 (1992). 10.1103/physrevb.46.9515 10002759

[52] A. T. Hatke, M. A. Zudov, J. D. Watson, M. J. Manfra, L. N. Pfeiffer, K. W. West, Evidence for effective mass reduction in GaAs/AlGaAs quantum wells. Phys. Rev. B 87, 161307 (2013).

[53] Y.-W. Tan, J. Zhu, H. L. Stormer, L. N. Pfeiffer, K. W. Baldwin, K. W. West, Measurements of the density-dependent many-body electron mass in two dimensional GaAs/AlGaAs heterostructures. Phys. Rev. Lett. 94, 016405 (2005). 15698108 10.1103/PhysRevLett.94.016405

[54] Y. Kwon, D. M. Ceperley, R. M. Martin, Quantum Monte Carlo calculation of the Fermi-liquid parameters in the two-dimensional electron gas. Phys. Rev. B 50, 1684–1694 (1994). 10.1103/physrevb.50.1684 9976356

