# Supplementary material for: Observing separate spin and charge Fermi seas in a strongly correlated one-dimensional conductor
Source: Sci Adv. 2022 Jun 17;8(24):eabm2781. doi: 10.1126/sciadv.abm2781 (PMC9205598; doi:10.1126/sciadv.abm2781)
Supplement: Supplementary file 1 — Supplementary Text Figs. S1 to S9 References [file sciadv.abm2781_sm.pdf]

Science Advances
AAAS

Supplementary Materials for

Observing separate spin and charge Fermi seas in a strongly correlated one-dimensional conductor

Pedro M. T. Vianez *et al.*

Corresponding author: Pedro M. T. Vianez, pmtv2@cam.ac.uk; Oleksandr Tsyplyatyev, o.tsyplyatyev@gmail.com; Christopher J. B. Ford, cjbf@cam.ac.uk

Sci. Adv. **8**, eabm2781 (2022)
DOI: 10.1126/sciadv.abm2781

This PDF file includes:

Supplementary Text
Figs. S1 to S9
References

1 Supplemental Text

1.1 Hubbard model

Interacting electrons in our quantum wires can be described by the 1D Fermi-Hubbard model,

$$H = -t \sum_{j=1,\alpha=\uparrow,\downarrow}^{L/a} \left(c_{j\alpha}^\dagger c_{j+1,\alpha} + c_{j\alpha}^\dagger c_{j-1,\alpha} \right) + U \sum_{j=1}^{L/a} n_{j\uparrow} n_{j\downarrow}, \tag{S1}$$

where $c_{j\alpha}$ are the second-quantisation operators in real space obeying the Fermi commutation relations $\left\{ c_{j\alpha}, c_{j'\alpha'}^\dagger \right\} = \delta_{jj'}\delta_{\alpha\alpha'}$, α is the spin-1/2 index $\uparrow$ or $\downarrow$, $n_{j\alpha} = c_{j\alpha}^\dagger c_{j\alpha}$ is the local density operator for each of the spin species, L is the length of the wire, and a is the lattice parameter of the host crystal. The two microscopic constants of the model are the hopping amplitude t, describing the kinetic energy, in the first term and the two-body interaction energy U in the second term. The local nature of the density-density interaction in this model allows for only a single microscopic constant U that takes into account at the same time the strength of the Coulomb interaction governed by the electronic charge e and the screening radius R, which we can tune in our experiment.

The many-body eigenstates of the Hubbard model in 1D were constructed in [40, 41]. They describe the amplitude of finding all N particles at a given set of sites on the lattice $j_1, \ldots, j_N = \boldsymbol{j}$ and with a given configuration of their spins $\alpha_1, \ldots, \alpha_N = \boldsymbol{\alpha}$: $\Psi = \sum_{\boldsymbol{j},\boldsymbol{\alpha}} a_{\boldsymbol{j}\boldsymbol{\alpha}} c_{j_1\alpha_1}^\dagger \cdots c_{j_N\alpha_N}^\dagger |0\rangle$. These amplitudes have the form of a superposition of plane waves,

$$a_{\boldsymbol{j}\boldsymbol{\alpha}} = \sum_P A_{PQ\boldsymbol{\alpha}} e^{i(P\boldsymbol{k}a)\cdot(Q\boldsymbol{j})}, \tag{S2}$$

where Q is the permutation that orders all N coordinates such that

$$Qj_1 < \cdots < Qj_N, \tag{S3}$$

the momenta of N particles are $\boldsymbol{k} = k_1, \ldots, k_N$, and $\sum_P$ is the sum over all permutations of the charge momenta, like in the Slater determinant for free particles. However, unlike for free particles, the sign under the permutation of a pair of coordinates is not -1 but is rather a phase factor $A_{PQ\boldsymbol{\alpha}}$ that also depends on the spin configuration $Q\boldsymbol{\alpha}$ as

$$A_{PQ\boldsymbol{\alpha}} = (-1)^{PQ} \sum_R \left(\prod_{1 \le l < m \le M} \frac{R\lambda_l - R\lambda_m - \frac{iU}{2t}}{R\lambda_l - R\lambda_m} \right) \times \prod_{l=1}^{M} \frac{\frac{iU}{2t}}{R\lambda_l - \sin Pk_l a + \frac{iU}{4t}} \prod_{j=1}^{s_l - 1} \frac{R\lambda_l - \sin Pk_j a - \frac{iU}{4t}}{R\lambda_l - \sin Pk_j a + \frac{iU}{4t}}, \tag{S4}$$

where $\boldsymbol{s} = s_1, \ldots, s_M$ are the coordinates of the M spins $\uparrow$ in the configuration $\boldsymbol{\alpha}$ of all spins of N particles, $\boldsymbol{\lambda} = \lambda_1, \ldots, \lambda_M$ are the spin momenta that correspond to these M spins $\uparrow$, and $\sum_R$ is the sum over all permutations of these spin momenta.

Spins of N electrons in a quantum wire form a spin chain since the positions of the electrons are ordered, permitting us to determine whether one particular spin is to the left or right of another. For example, let us consider a spin configuration for $N = 8$ electrons, of which 5 spins are $\downarrow$ and 3 spins are $\uparrow$,

$$\alpha_1 \ldots \alpha_N = \downarrow\downarrow\uparrow\downarrow\uparrow\uparrow\downarrow\downarrow . \tag{S5}$$

Here the positions of spins $\uparrow$ are $\boldsymbol{s} = 3, 5, 6$, which can be interpreted as coordinates of $M = 3$ spin excitations in the spin chain of length $N = 8$. These spin positions can change when electrons of opposite spin interact with each other via the Coulomb interaction, changing their coordinates in the wire and making the spin eigenmodes extended, at least at low densities away from the Mott-insulator regime. Therefore, M spin excitations are characterised by M spin momenta, like the charge particles. Moreover, electrons with the same momenta can also interact, making their spins interact with each other and causing the many-spin states consisting of M individual spins to be correlated in their own right.

The momenta are quantised by boundary conditions. Application of the periodic boundary condition to the many-particle wave function in Eq. (S2) gives the Lieb-Wu equations [22],

$$k_j L - \sum_{m=1}^{M} \varphi\left(\lambda_m - k_j a\right) = 2\pi I_j, \tag{S6}$$

$$\sum_{j=1}^{N} \varphi\left(\lambda_m - k_j a\right) - \sum_{l=1}^{M} \varphi\left(\lambda_m/2 - \lambda_l/2\right) = 2\pi J_m, \tag{S7}$$

$$\text{with} \quad \varphi\left(x\right) = -2\arctan\left(\frac{4tx}{U}\right), \tag{S8}$$

where N non-equal integers I_j and M non-equal integers J_m define the solution for the orbital k_j and the spin λ_m momenta of an N-electron state for a given value of the microscopic parameter U/t. This solution also gives the eigenenergy of the many-electron state as $E = ta^2 \sum_{j=1}^{N} k_j^2$ and its momentum as $k = \sum_{j=1}^{N} k_j$. These simultaneous quantisation conditions for both spin and charge degrees of freedom are a system of $N + M$ connected nonlinear equations for any finite U.

1.2 Two Fermi seas

Both the charge and the spin momenta correspond to non-equal integer numbers I_j and J_m since the 1D wave function in Eq. (S2) becomes zero for any pair of equal k_j or λ_m, which are obtained immediately from Eqs. (S6, S7) when a pair of I_j or J_m are equal. In principle, such an emergent picture is described by two different Fermi seas, in which both kinds of modes are

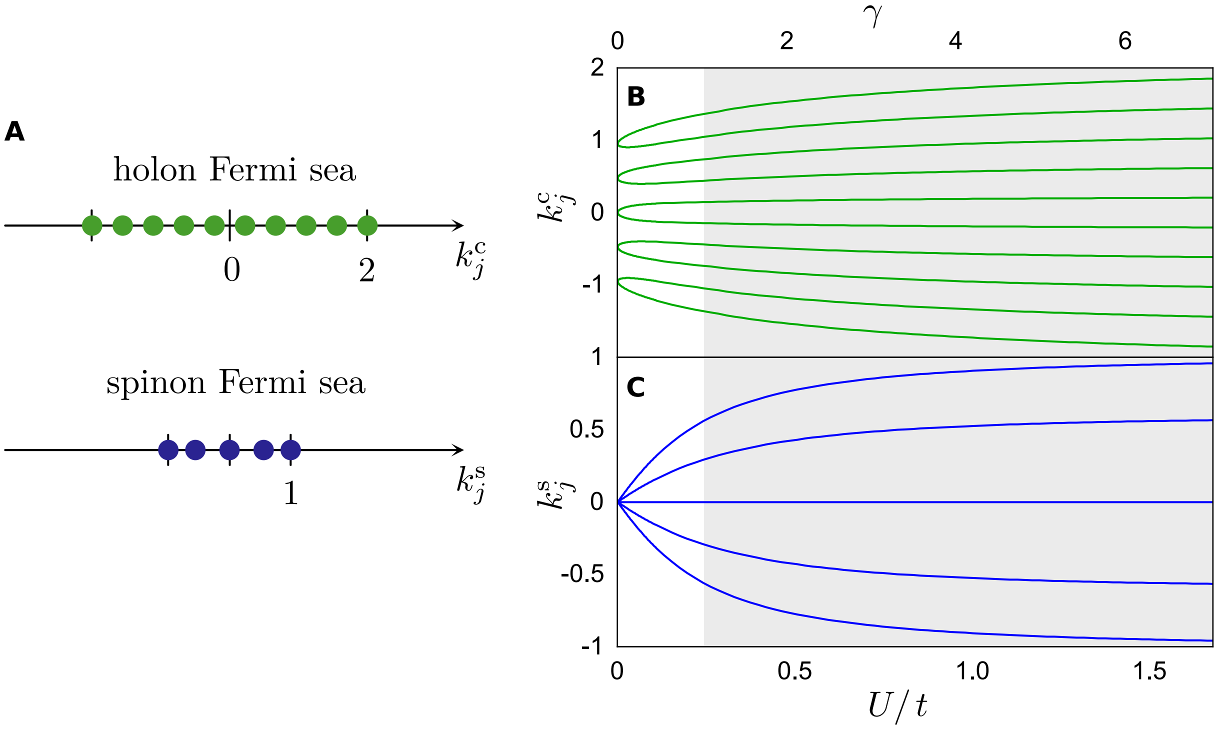

Figure S1: **Ground state of the Fermi-Hubbard model.** (**A**) Solutions of the Lieb-Wu equations (S6) and (S7) for the charge and the spin degrees of freedom for the ground state in the infinite interactions limit $U = \infty$ and its evolution for different values of U for the holon (**B**) and the spinon (**C**) Fermi seas for $N = 10$ unpolarised $M = 5$ electrons. The charge momenta are normalised by the free-electron Fermi momentum, $k_j^c = k_j/k_F$ where $k_F = \pi N/(2L)$ and the spin momenta $k_j^s = -2\arctan[U/(4t\lambda_j)]/\Lambda$ are normalised by the solution of the integral Orbach equation Λ [42] that plays the role of the Fermi momentum for Heisenberg spin chains. The dimensionless parameter γ is defined in Eq. (S9). In the grey area, $\gamma > 1$, the interactions are strong enough for the two Fermi seas to be fully developed.

filled with spin or charge excitations up to some finite densities, just as the usual Fermi sea is filled with non-interacting fermions up to a finite Fermi energy. The difference for the problem with interactions is a more complicated relation between the two types of the occupation numbers, visualised by a set of I_j and J_m, and the actual distributions of the two types of momenta, k_j and λ_m. This relation is not just a linear quantisation condition for each momentum of the free particles independently but is rather given by the solution of the system of essentially non-linear coupled equations (S6) and (S7) for any finite interaction U. For instance, the quantisation conditions given by the Lieb-Wu equation in Eqs. (S6, S7) alter qualitatively the equilibrium properties by changing parameters of the two Fermi seas or by changing the excitation energies of different modes, making their dispersions generally interaction-dependent.

Our quantum wires remain unpolarised up to the maximum field of 7 T that we use in our experiments. The number of spin-up and down electrons is equal within 10% (5% below B_+),

and we therefore assume $M = N/2$ spin particles. For the holon Fermi sea, which has twice as many filled states as the spinon Fermi sea, the Lieb-Wu equations give the two distributions of their momenta at the infinite-interaction point $U = \infty$, see Fig. S1A. We define the Fermi momentum k_{F} by the free electrons, *i.e.* by $N/2$ fermions that are doubly degenerate with respect to spin without the interactions at $U = 0$. For strong enough interactions the free-electron degeneracy is completely lifted, increasing the density of the charge particles by the factor of two. At the same time, the spin particles develop the second Fermi sea of the Heisenberg-spin-chain type [43] with a density that is twice as small as that of the holons. The momentum distribution in the spinon Fermi sea is non-equidistant: the spin momenta have a pronounced higher density towards the Fermi points but are sparser at the bottom of the band [44]. A Fermi sea of this kind was observed using neutron scattering in non-itinerant magnets [30, 31, 32].

Decreasing the interaction energy U away from the infinite point, we solve the Lieb-Wu equations (S6) and (S7) numerically, see Figs. S1B and C. Down to an intermediate value of U both Fermi seas remain stable, but below it the two-seas picture starts to change qualitatively. For weak interactions the holon Fermi sea becomes doubly degenerate, recovering the free-fermion picture, and the spinon Fermi sea collapses, becoming the spin part of the free-electron function that describes ± 1 permutation signs for the electrons of different or the same spin.

The dimensionless parameter γ, which controls this transition, emerges from the Hubbard model itself microscopically. It was identified in a quantitative analysis of the double occupancy of the electronic states [37] as

$$\gamma = \frac{\lambda_{\mathrm{F}}}{16a} \frac{U}{t} \frac{1}{1 - \frac{1}{N} \sum_{l=1}^{N/2} \frac{\lambda_l^2(\infty) - \left(\frac{U}{4t}\right)^2}{\lambda_l^2(\infty) + \left(\frac{U}{4t}\right)^2}}, \tag{S9}$$

where $\lambda_{\mathrm{F}} = 4L/N$ is the Fermi wavelength of the free-electron gas, a is the lattice parameter, and $\lambda_l(\infty)$ are the spin part of the solution of Eqs. (S6) and (S7) in the infinite-interaction limit $U = \infty$. Turning the sum into an integral in the thermodynamic limit and evaluating it for the unpolarised Heisenberg chain [42] we obtain the numerical value of the denominator in the above expression, $1 - \sum_l \ldots /N = 1.193(1)$. Thus, in the thermodynamic limit the parameter controlling the interaction effects is

$$\gamma = 0.032 \frac{\lambda_{\mathrm{F}}}{a} \frac{U}{t}. \tag{S10}$$

When the interactions are strong, $\gamma > 1$, both the spinon and the holon Fermi seas are fully developed but, when $\gamma < 1$, the holon Fermi sea is still close to the double occupancy of the free Fermi gas. The expression in Eq. (S10) is presented in Eq. (1) in the main text.

1.3 Spin and charge excitations

By adding a single electron with its charge and spin we add two excitations to the system simultaneously, one holon and one spinon. With two Fermi seas there are two options. One

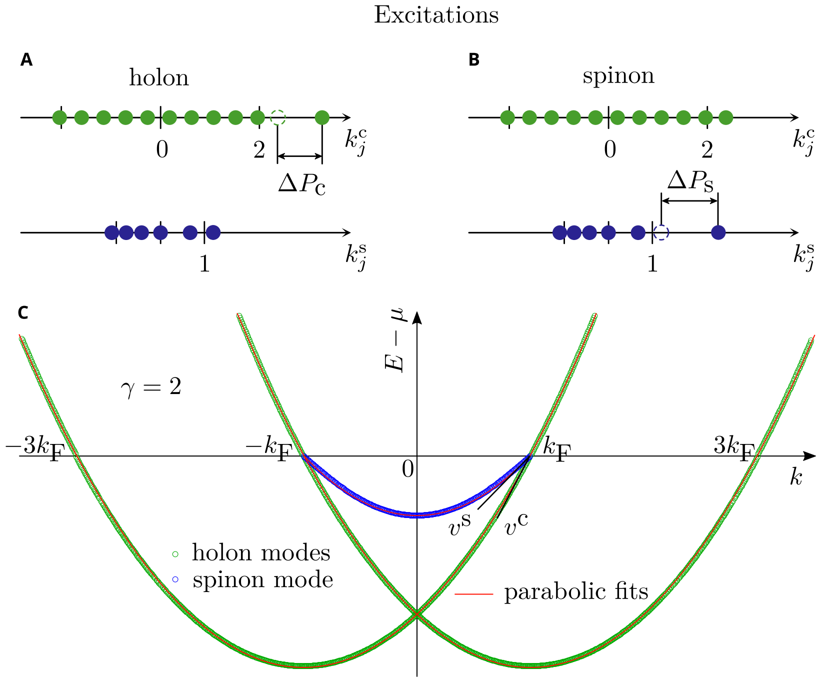

Figure S2: **Spinon and holon excitations of Fermi-Hubbard model.** (**A**) Excitation of the holon type, in which the spinon part is placed at the lowest possible momentum for the spin excitations and the holon part is promoted to some finite momentum, which is larger than the lowest possible momentum for the charge excitations by $\Delta P_{\rm c}$. (**B**) Excitation of the spinon type, in which the holon part is placed at the lowest possible momentum for the charge excitations and the spin part is promoted to some finite momentum, larger than the lowest possible momentum for the spin excitations by $\Delta P_{\rm s}$. (**C**) The dispersions of the holon (green circles) and of the spinon (blue circles) modes consisting of the excitations in (**A**) and (**B**), respectively, obtained by solving the Lieb-Wu equations (S6, S7) for $N = 550$ unpolarised electrons with $M = 275$ for interaction strength $\gamma = 2$. The red lines are the parabolic fits for both modes. The two black lines at the Fermi point $k_{\rm F}$ mark the linear dispersions of holons $v^{\rm c}$ and of spinons $v^{\rm s}$ of the linear spinful Tomonaga-Luttinger model.

is adding a holon to its Fermi sea at the $2k_{\rm F}$ point and spinon to its Fermi sea at $-k_{\rm F}$ point on top of the ground state in Fig. S1A, obtaining the net momentum for the electronic excitation $k = k_{\rm F}$. Another possibility is adding the holon and the spinon at the same sides of their Fermi seas, producing the net momentum $k = 3k_{\rm F}$. Our wires have inversion symmetry. Therefore, the mirror of this argument produces the $k = -k_{\rm F}$ and the $k = -3k_{\rm F}$ as well, see Fig. S2C.

Starting at a Fermi point, say $k_{\rm F}$, we can add a pair of excitations of the two kinds not just at the Fermi points of their respective Fermi seas but also at some finite momenta, $\Delta P_{\rm c}$ or $\Delta P_{\rm s}$, above them, see Fig. S2A and B. Change of the momentum of any of these excitations distorts both Fermi seas via solving the nonlinear Lieb-Wu equations (S6) and (S7): all states in the spinon Fermi sea become generally asymmetric, in addition to being non-equidistant in the ground state, and the holon Fermi sea as a whole shifts by the total spinon momentum, see Figs.

S2A and B. However, the total momentum of the electronic excitation is a quantum number, which has a simple relation, $k = 2\pi \left(\sum_j I_j + \sum_m J_m\right)/L$, to the whole set of the quantisation integer numbers, I_j and J_m, and is independent of U [22]. This result of the total momentum conservation law for a many-body system defines the momentum of the holon and the spinon excitations as

$$k = k_{\mathrm{F}} + \Delta P_{\mathrm{c}} \quad \text{and} \quad k = k_{\mathrm{F}} + \Delta P_{\mathrm{s}}, \tag{S11}$$

where $\Delta P_{\mathrm{c}} = 2\pi I_{N+1}/L$ and $\Delta P_{\mathrm{s}} = 2\pi J_{M+1}/L$ can be visualised in a simple way due to the same total momentum conservation for all particles. Therefore, the distance in the momentum variable between the neighbouring points is always equal to the quantum of momentum $2\pi/L$ and the position of the second crossing points of the holon modes with the line of chemical potential in Fig. S2C always remains at the same momentum $\pm 3k_{\mathrm{F}}$, irrespective of the value of U.

The energies of these excitations, on the other hand, are more complicated. In order to evaluate them and the dispersions that they form, we need to solve the Lieb-Wu equations (S6) and (S7) numerically for different values of U, see Fig. S2C. At low energies, the solutions form two linear dispersions with non-commensurate slopes, v^{c} and v^{s}; the stronger U is, the larger is the ratio $v^{\mathrm{c}}/v^{\mathrm{s}}$, and the two velocities become equal $v^{\mathrm{c}}/v^{\mathrm{s}} = 1$ in the free-particle limit $U = 0$. These two holon and spinon velocities are nothing but a pair of the Luttinger parameters in the spinful linear Tomonaga-Luttinger model [45, 24] at low energies. They are manifested in the observable spectral function as a pair of divergences [26, 27].

Extensions of the momenta of the excitations of two different kinds, ΔP_{c} and ΔP_{s}, away from the $\pm k_{\mathrm{F}}$ points provide a natural generalisation of these linear modes to the nonlinear regime, see Fig. S2C. Unlike the linear regime, where there are only two branches around each of the Fermi points, in the whole energy band there are three. This is a manifestation of the different densities of the holon and the spinon Fermi seas. Dispersions of all of the three curves are not exactly parabolic for finite U but are close to parabolae due to an interaction effect between the two Fermi seas, with the most significant deviations occurring for the spinon branch in the vicinity of the Fermi points. Fitting parabolae to the numerically produced exact dispersions (red lines in Fig. S2C), gives the dependence of the two masses m_{c} and m_{s} on the interaction parameter γ. Note that for a finite $\gamma > 0$ these two masses are non-commensurate but they become commensurate, $m_c = 2m_s$, in the $\gamma = 0$ limit. The ratio of these masses is presented in Fig. 4B of the main text.

The two velocities v^{c} and v^{s} are extracted as the linear coefficients in the dispersion of the holon and the spinon modes at the Fermi energy, and the dependence of their ratio on γ is also presented in Fig. 4B. We fit our experimental data with the exact dispersions of the Hubbard model to obtain the interaction parameter γ directly, and also with the two parabolae, see more details in the section 'Parabolic Model' below.

1.4 Mode hierarchy away from the Fermi points

This section follows the work in [19, 20] previously described in [46]. A systematic understanding of the general picture for nonlinear excitations has come from a microscopic analysis of the spinless counterpart of the 1D Fermi-Hubbard model. Using the analysis of the correlation functions via the algebraic Bethe ansatz, it was found [19, 20] that away from the Fermi points the exponentially many excitations forming the many-body continuum are separated into levels of a mode hierarchy according to their spectral strength, which is proportional to integer powers of a small parameter R^2/L^2, where R is the radius of the two-body interaction potential and L is the length of the system. A detailed analysis of these amplitudes revealed that the strongest excitations (with the zeroth power of R^2/L^2) form a parabola-like dispersion, which corresponds to the solid green line in Fig. S3, similar to that of the original non-interacting fermions but with some renormalisation due to interactions. All other many-body modes have powers of R^2/L^2 greater than zero, with the general trend of increasing the integer by one with each discrete step of $2k_{\mathrm{F}}$ along the k-axis away from the principal parabola, see the dashed lines in Fig. S3 and their labelling scheme in the caption to the figure.

As an example, the hole part of the principal parabola, between the $\pm k_{\mathrm{F}}$ points, has the largest amplitude but its mirror in the particle sector—a 'replica' in the shape of a dome marked as $p0b$ by the dashed green in Fig. S3—has a parametrically smaller amplitude, proportional to the first power of R^2/L^2. In the spectral function the strength of this replica is predicted to be

$$A_1(k,E) \propto \frac{R^2}{L^2}\frac{k_{\mathrm{F}}^2 k^2}{\left(k^2-k_{\mathrm{F}}^2\right)^2}\delta\left(E-\mu+\xi_1(k)\right), \tag{S12}$$

where $\xi_1(k)$ is the dispersion for the 'replica'. Therefore, for almost all momenta this mode will be unobservable in the thermodynamic limit. The only exceptions are the regions around the $\pm k_{\mathrm{F}}$ points, where the singularity in the denominator starts to compete with the parametric smallness, resulting in a large amplitude overall. On the other hand, the measurement of the whole mode requires using smaller systems, in which the R^2/L^2 parameter still leaves the amplitude of the whole mode above the background from the other processes.

Close to the spectral threshold the mode hierarchy reproduces the predictions of the mobile-impurity model. The small parameter R^2/L^2 provides a path for accounting for the principal amplitudes of the exponential continuum of many-body excitations, which leads to the microscopic calculation of the spectral function. The resulting threshold exponents match those predicted by the phenomenologically introduced nonlinear hydrodynamic model.

While the mode hierarchy emerges away from the Fermi points, close to them (where the spectrum is almost linear) it transitions into the usual linear TLL, see details in [20]. The hydrodynamic modes of the latter consist of a huge number of many-body modes, all of which are of similar spectral strength, making the two regimes distinct already on the microscopic level. The change from one into the other can be traced quite easily using a macroscopic quantity, the density of states. It can be calculated exactly by numerical means using the Bethe ansatz approach, exhibiting the power-law suppression around the Fermi energy E_{F} predicted by the

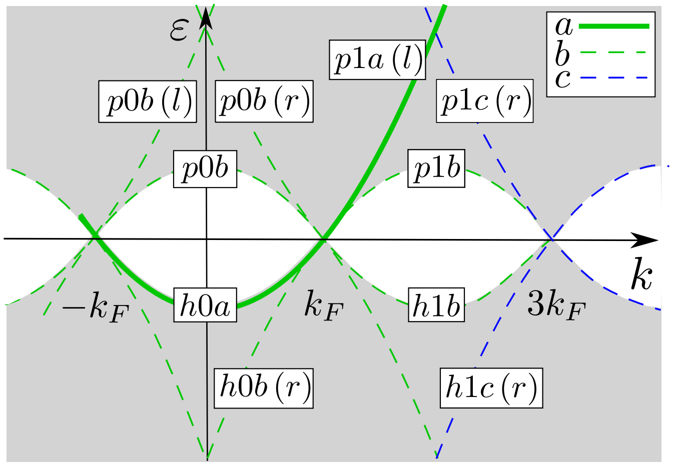

Figure S3: **Spectral function of spinless fermions according to the mode-hierarchy picture.** First (second)-level modes are shown in the region $-k_\mathrm{F} < k < k_\mathrm{F}$ ($k_\mathrm{F} < k < 3k_\mathrm{F}$) and are labelled by 0 (1), where k_F is the Fermi wave vector. Accessible and forbidden regions are marked by grey and white, respectively. Particle (hole) sectors are marked by p (h) for positive (negative) energies, a, b and c correspond respectively to the level in the mode hierarchy in powers of 0, 1 and 2 of R^2/L^2 and (r, l) correspond to the origin in the range, right or left.

TLL model and a crossover into a finite density $\propto 1/\sqrt{E}$ predicted by the mode hierarchy away from the linear region, where the nonlinearity of the single-particle dispersion already destroys the hydrodynamic modes of the TLL.

In our experiment, each mode of the hierarchy should, in principle, be split into two by the effect of spin-charge separation. We start observing the spin part of this p0b 'replica' mode in wires of 5μm and shorter lengths, see region C in Fig. 3A and Fig. 3C in the main text, whereas the p1b replica, see region B in Fig. 3A, is visible at 5μm, and strengthens as the length of the wire decreases, as shown in Fig. 3B in the main text.

1.5 Interaction parameter r_s in 1D

The Wigner-Seitz radius r_s is used as a generic interaction parameter in Fermi systems independent of their dimensionality. Its common applicability stems from the fact that the Fermi energy is inversely proportional to the inter-particle distance squared and that the Coulomb energy is inversely proportional to the same inter-particle distance but to the first power. Therefore, the dimensionless ratio of the interaction energy to the kinetic energy is just given by r_s.

We can extract the value of the Fermi wavelength λ_F in our experiment from the density of the 1D system, which can be obtained from either the 1D wire subbands dispersion (*e.g.* see Fig. S4) or alternatively by using the zero-field intersect points in the tunnelling conduction maps (see section on 'Parabolic Model'). This gives us two independent estimates for the electron density. Its relation to r_s in 1D is as follows. In one dimension the electron density is expressed

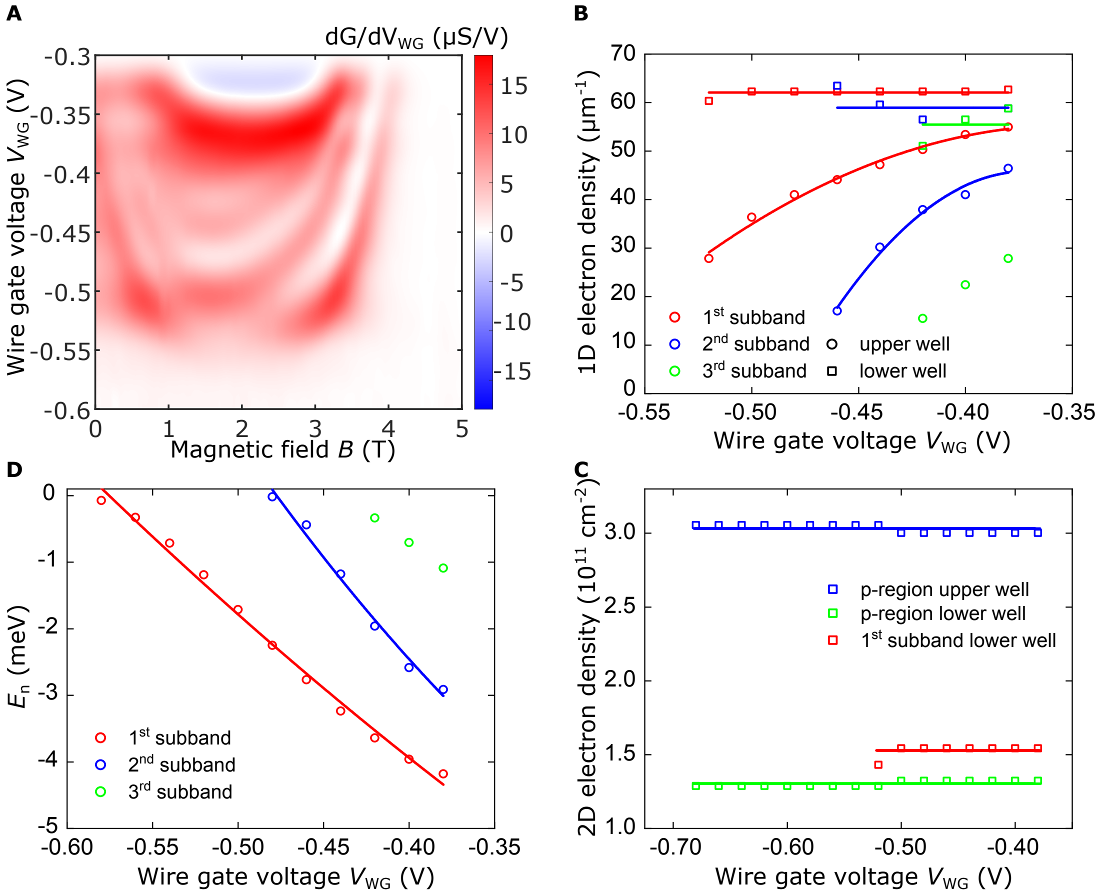

Figure S4: **1D wire subbands.** (**A**) $\mathrm{d}G/\mathrm{d}V_{\mathrm{WG}}$ differential of the tunnelling conductance G with respect to the wire-gate voltage V_{WG}, as a function of V_{WG} and magnetic field B perpendicular to the wires, as obtained under equilibrium conditions $V_{\mathrm{DC}} = 0$, for a 1.7 μm device. Three fully developed 1D subbands can be seen below the 2D band at $V_{\mathrm{WG}} \approx -0.35\,\mathrm{V}$ and above the cutoff point at $V_{\mathrm{WG}} \approx -0.55\,\mathrm{V}$, below which the wires cannot conduct. (**B**) Equilibrium 1D electron densities in both upper (circles) and lower (squares) wells for each occupied subband. Note that the electrons are not laterally confined in the bottom well and as such the lower well 1D density has no physical meaning and is only shown for comparison. (**C**) 2D electron densities in both upper (blue) and lower (green) wells' 'parasitic' regions. The overall independence of the 2D densities on the wire-gate voltage and the proximity of the (now physical) 2D electron density of the bottom well below the wire region (red) to that of the injection region shows that the bottom 2DEG remains largely unaffected by V_{WG}, and as such can be taken to be a well-understood 2D probe. (**D**) Subband energies relative to the chemical potential $\mu = 0$ as a function of V_{WG}.

in terms of r_{s} as

$$n = \frac{1}{2 a_{\mathrm{B}} r_{\mathrm{s}}}, \tag{S13}$$

where

$$a_{\mathrm{B}} = \frac{4\pi\varepsilon\varepsilon_0\hbar^2}{me^2} \tag{S14}$$

is the Bohr radius of electrons in GaAs, with $\varepsilon \approx 12$ and $m = 0.067m_{\mathrm{e}}$. On the other hand, the density of free electrons in a 1D Fermi sea is also given by the integral over the Fermi function,

$$n = 2\int \frac{dk}{2\pi} n_k = \frac{4}{\lambda_{\mathrm{F}}}, \tag{S15}$$

where the occupation numbers are a step function, $n_k = \theta\left(k_{\mathrm{F}} - |k|\right)$, and $k_{\mathrm{F}} = 2\pi/\lambda_{\mathrm{F}}$. Combining Eqs. (S13) and (S15) produces the following relation

$$r_{\mathrm{s}} = \frac{\lambda_{\mathrm{F}}}{8a_{\mathrm{B}}}. \tag{S16}$$

This generic dimensionless interaction parameter r_{s} plays the same role as the microscopic dimensionless interaction parameter of the 1D Fermi-Hubbard γ in Eq. (S9). However, r_{s} does not account for screening effects, see the main text.

The 1D subband structure of electrostatically-gated wires such as the ones used in this experiment can be observed from Fig. S4A. As can be seen, this is relatively simple, with our measurements being in very good agreement with the predictions and simulations of [47] and [48]. In specific, [48] took into account the explicit effect of e-e interactions into the (sub)band structure to some extent, finding no significant change in the overall structure. This, together with an observed subband spacing of $\sim 2\,\mathrm{meV}$ (see also Fig. S4D), allows us to individually populate each subband which should, therefore, not strongly hybridise. This simple (sub)band structure is in full agreement with what is observed in the tunnelling maps, both when in the 2D-2D and 1D-2D regimes, see section 1.6. for more details.

1.6 Parabolic model

When fitting the 1D tunnelling resonances observed in the dispersion maps, one can approximate the exact numerical solutions arising from the Hubbard model by parabolae, see Supplementary Text 'Spin and charge excitations' and Fig. S2C for both spin- or charge-type excitations. On the other hand, 2D systems at low densities (see Fig. S4C), are known to be Fermi liquids with effective mass renormalised by interactions. Together these account for the dispersion of the elementary excitations in both the upper and lower wells (UW and LW), which can behave as either 2D or 1D systems depending on whether the signal arises from unconfined ('parasitic'-, LW) or confined (wire-) regions of the device. We will now show how separation between spin- and charge-type modes in 1D, each associated with different effective masses and degeneracies, emerges naturally from the data after considering simpler models. We start by introducing the capacitive-coupling correction, which was also applied when fitting using the Hubbard model.

1.6.1 Modelling the capacitive coupling

Tunnelling between the upper and lower layers of a device, across its dielectric barrier, is affected by capacitance effects, which result in the observed dispersions being slightly asymmetric. Owing to the finite capacitance C between the two layers, a small increase or reduction of the electron density $\pm\delta n_{\mathrm{2D/1D}}$ occurs at each side of the barrier. We have $e\delta n_{\mathrm{2D}} = V_{\mathrm{DC}}C/A$ for the 2D system (of area A) and $e\delta n_{\mathrm{1D}} = V_{\mathrm{DC}}C/L$ for the corresponding 1D system (of total length L), with V_{DC} the DC-bias applied between the wells. For two layers with Fermi wavevectors $k_{\mathrm{F},1}$ and $k_{\mathrm{F},2}$, where $k_{\mathrm{F},1} < k_{\mathrm{F},2}$, the zero-bias crossing points B_+ and B_- can be combined to give

$$k_{\mathrm{F},1} = \frac{ed}{2\hbar}(B_+ - B_-), \qquad k_{\mathrm{F},2} = \frac{ed}{2\hbar}(B_+ + B_-), \tag{S17}$$

where d is the separation between the wells. Our 1D wires, once defined by the gate voltage, do indeed have lower density than the 2D system beneath, so in the wire region we take $k_{\mathrm{F},1}$ as the Fermi wavevector of the 1D system in the absence of interactions, and $k_{\mathrm{F},2}$ as that of the 2D layer.

The electron densities for 1D and 2D systems are given by

$$n_{\mathrm{1D}} = \frac{\mathrm{d}N}{\mathrm{d}L} = \frac{2k_{\mathrm{F,1D}}}{\pi} \tag{S18}$$

and

$$n_{\mathrm{2D}} = \frac{\mathrm{d}N}{\mathrm{d}A} = \frac{k_{\mathrm{F,2D}}^2}{2\pi}. \tag{S19}$$

From here, we get

$$\delta n_{\mathrm{1D}} = n_{\mathrm{1D}}(V_{\mathrm{DC}}) - n_{\mathrm{1D}}(0) = \frac{\eta_i C V_{\mathrm{DC}}}{eL} = \frac{2}{\pi}(k_{\mathrm{F,1D}}(V_{\mathrm{DC}}) - k_{\mathrm{F,1D}}(0)) \tag{S20}$$

and

$$\delta n_{\mathrm{2D}} = n_{\mathrm{2D}}(V_{\mathrm{DC}}) - n_{\mathrm{2D}}(0) = \frac{\eta_i C V_{\mathrm{DC}}}{eA} = \frac{1}{2\pi}(k_{\mathrm{F,2D}}^2(V_{\mathrm{DC}}) - k_{\mathrm{F,2D}}^2(0)), \tag{S21}$$

which in turn gives the modified Fermi wavevectors as the inter-layer voltage V_{DC} causes the densities to change,

$$k_{\mathrm{F,1D}}(V_{\mathrm{DC}}) = k_{\mathrm{F,1D}}(0) + \frac{\pi\eta_i C V_{\mathrm{DC}}}{2eL} \tag{S22}$$

and

$$k_{\mathrm{F,2D}}(V_{\mathrm{DC}}) = \sqrt{k_{\mathrm{F,2D}}^2(0) + \frac{2\pi\eta_i C V_{\mathrm{DC}}}{eA}}. \tag{S23}$$

Here $\eta_i = \pm 1$, with $i = 1, 2$ labelling the upper/lower layer respectively, is a sign factor based on the experimental setup. In our experiment, $\eta_1 = -1$ and $\eta_2 = 1$, since for $V_{\mathrm{DC}} > 0$ the upper well was more positive than the lower well.

The bottom well is always 2D in nature, which means that $k_{\mathrm{F},2} = k_{\mathrm{F,2D}}(V_{\mathrm{DC}})$ with $\eta_2 = 1$. The top well, on the other hand, can behave as being either 2D, in the injection region, or 1D, in

the wires. For the latter we have $k_{\mathrm{F},1} = k_{\mathrm{F,1D}}(V_{\mathrm{DC}})$ with $\eta_1 = -1$. For the former, the upper-well density is higher than in the lower well. Note also that equation (S23) reduces to equation (S22) when expanded in the low-capacitance limit,

$$k'_{\mathrm{F,2D}} = k_{\mathrm{F,2D}}\left(1 + \frac{2\pi\eta_i C V_{\mathrm{DC}}}{eAk^2_{\mathrm{F,2D}}}\right)^{1/2} \tag{S24}$$

$$\approx k_{\mathrm{F,2D}} + \frac{\pi\eta_i C V_{\mathrm{DC}}}{eAk_{\mathrm{F,2D}}}. \tag{S25}$$

Therefore, comparing Eqs. S22 and S25 we conclude that the capacitance correction for the wire region, which is 1D in nature, can nevertheless be treated as for a 2D system by transforming the capacitance as $C/L = C/A \times 2/k_{\mathrm{F,1D}}$. One can also assume charge neutrality of the pair of layers to estimate the width of the wires w by solving $C/L = w \times C/A$.

We now estimate the capacitive coupling between the two wells in our setup, when applying a voltage to the upper layer and keeping the potentials of the gates and lower layer fixed. This is required to correct for the mapped dispersions as discussed previously. In a classical Coulomb system, the capacitance $C \equiv \mathrm{d}Q/\mathrm{d}V_{\mathrm{DC}}$ of a conductor is a purely geometric quantity (for charge Q on the conductor). For example, in a parallel-plate capacitor, it takes the well-known value $C = \varepsilon\varepsilon_0 A/d$, A being the surface area of the plates, d their separation, and ε the relative permittivity of the dielectric material in between. In systems with a low density of states however, such as a 2DEG, this result does not strictly apply, since unlike for a perfect metal plate, the density of states here is no longer infinite. Therefore, in contrast to a metal, a 2DEG cannot, in general, perfectly screen the electrical field generated by the surface gates.

In order to account for this effect one has to consider band-filling/band-emptying in changing the density of states in the 2DEG as the gate voltage is varied. This correction was initially proposed in [49] and can be modelled by considering two capacitors connected in series,

$$\frac{1}{C} = \frac{1}{C_{\mathrm{G}}} + \frac{1}{C_{\mathrm{Q}}}, \tag{S26}$$

where C_{G} is the usual geometric capacitance, $C_{\mathrm{Q}} = e^2 dn_{\mathrm{2D}}/dE_{\mathrm{F}}A$ is the new quantum capacitance, and E_{F} is the Fermi energy as measured relative to the bottom of the band and thus varies as the occupation changes. Depending on dimensionality, the ratio of the two capacitances is then given by

$$\frac{C_{\mathrm{G}}}{C_{\mathrm{Q}}} = \begin{cases} \dfrac{\hbar^2\pi^2\varepsilon\varepsilon_0 w}{4m^* e^2 D} n_{\mathrm{1D}} & \text{for gate-wire system,} \\ \dfrac{\hbar^2\pi\varepsilon\varepsilon_0}{m^* e^2 D} & \text{for gate-2DEG system,} \end{cases} \tag{S27}$$

where D is the distance from the wells to the surface, w is the width of the wire, and m^* is the electron effective mass in GaAs. Here, we assumed $n_{\mathrm{1D}} = n_{\mathrm{2D}} \cdot w$, with n_{1D} and n_{2D} as defined in equations (S18) and (S19), respectively. Taking $w = 50\,\mathrm{nm}$, $D = 85\,\mathrm{nm}$, $m^* = 0.067m_{\mathrm{e}}$, $\varepsilon = 12$ and $n_{\mathrm{1D}} = 33\,\mu\mathrm{m}^{-1}$, we get $C_{\mathrm{G}}/C_{\mathrm{Q}} = 0.036$ and $C_{\mathrm{G}}/C_{\mathrm{Q}} = 0.028$ for a 1D and 2D

system, respectively. In both cases $C_{\mathrm{Q}} \gg C_{\mathrm{G}}$ and so the geometric contribution is expected to dominate in any capacitance measurement.

Our system consists of a GaAs/AlGaAs double-quantum-well heterostructure with translational invariance along the x- and y-directions, the wells being at roughly 70 and $100\,\mathrm{nm}$ below the surface. A set of surface gates is used to define the quantum wires in the upper well. There is capacitive coupling between the wells and also between each well and the surface gates, which provide screening to the 2D electron gas.

We used the COMSOL Multiphysics 5.5 software package at `www.comsol.com` to simulate our device electrostatically (not self-consistently) and computed the electrical field and the potential distribution in the dielectrics given the known charge distributions in each well, one of which had wires defined by the surface gates (see Fig. S4B and S4C). Specifically, when solving Poisson equation we took $U(\mathbf{r})$ as the potential induced by the gates and solved for it by taking $U(\mathbf{r}) = V_{\mathrm{SG}}$ at the gates and Neumann boundary conditions otherwise. In our simulation we accounted for the finite width of the 2D electron systems, though this made little difference to the results. Poisson's equation was solved using a finite-element grid, the number of nodes chosen so that the computation was free from finite-element effects. Finally, in our model, we ignored the effect of the ionised donor layers on both sides of the wells since they form a static layer of charge which is not affected by changes to gate voltage. The capacitance values (per unit area) obtained were $c = 0.0047\,\mathrm{Fm}^{-2}$ for the 2D injection region and $c = 0.0124\,\mathrm{Fm}^{-2}$ for the 1D wire region in the single-subband regime.

1.6.2 2D-2D tunnelling signal

In order to subtract the influence of the 'parasitic' injection region from the overall signal we map it beforehand by setting V_{WG} negative enough that the wires do not conduct (see Fig. S5, below the bottom subband). The parameters of interest when fitting in this regime are, respectively, the capacitances (per unit area) of both the upper and lower wells, $c^{\mathrm{2D}}_{\mathrm{UW}}$ and $c^{\mathrm{2D}}_{\mathrm{LW}}$, the effective masses of electrons in top and bottom wells, m_{UW} and m_{LW}, the separation between the wells, d, and the zero-bias field intercepts, B^{p}_{-} and B^{p}_{+}. Given that this region is relatively wide ($\sim 0.45\mu$m), it can be safely treated as a 2D system in both wells.

We found that good results were obtained when setting both m_{UW} and m_{LW} to be equal $0.93m_{\mathrm{b}}$, where $m_{\mathrm{b}} = 0.067m_{\mathrm{e}}$ is the electron band mass in GaAs. We note that $m^{*}_{\mathrm{2D}} = 0.93m_{\mathrm{b}} = 0.062m_{\mathrm{e}}$ is in very good agreement with independent work carried out in 2D systems very similar to ours [50, 51, 52, 53, 54]. From the MBE growth data we also know that $d \approx 32\,\mathrm{nm}$ and, even though it is reasonable to expect deviations from this value due to monolayer fluctuations, these should not exceed a few nanometres. Finally, the zero-bias crossing points can be easily determined by visual inspection of the data while the capacitive coupling was estimated by simulating it under an electrostatic framework following the approach described in the previous section. We found that the best results were obtained with $d = 31\,\mathrm{nm}$, $c^{\mathrm{2D}}_{\mathrm{UW}} = 0.0047\,\mathrm{Fm}^{-2}$ and $c^{\mathrm{2D}}_{\mathrm{LW}} = 0.0033\,\mathrm{Fm}^{-2}$, in very good agreement with the expected values. Owing to extra coupling to the surface gates, the calculated and observed capacitances of the

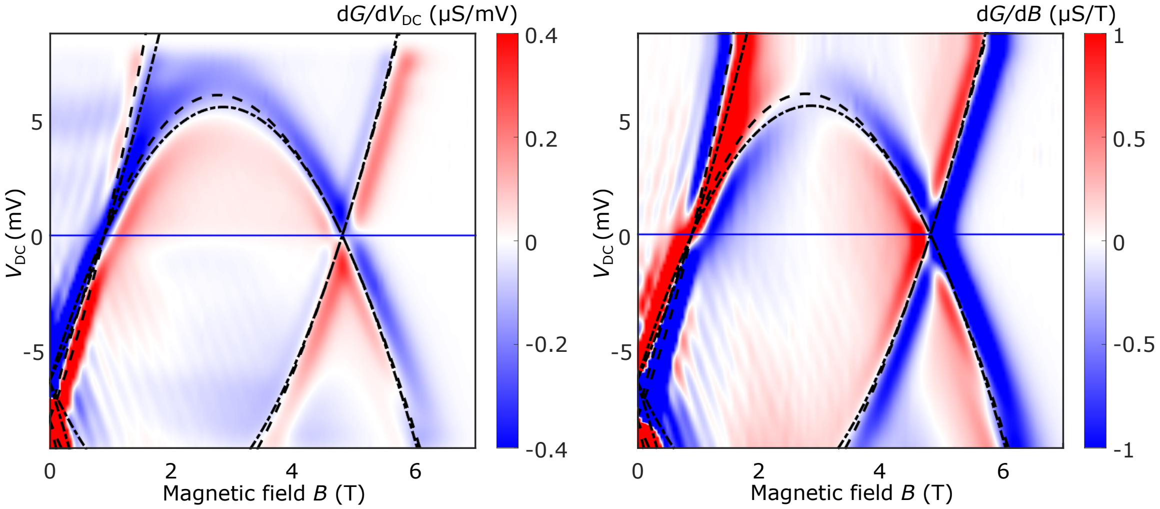

Figure S5: **2D-2D Background.** Tunnelling conduction differentials $\mathrm{d}G/\mathrm{d}V_{\mathrm{DC}}$ and $\mathrm{d}G/\mathrm{d}B$ vs magnetic field B ($\propto$ momentum) and voltage V_{DC} ($\propto$ energy eV_{DC}) for a $1.7\,\mu$m long device, mapped at $V_{\mathrm{WG}} = -0.57\,$V. At this voltage the wires are pinched off and do not conduct. Therefore, the spectrum observed arises solely from 2D-2D tunnelling taking place between the 'parasitic' injection region and the bottom 2DEG, with the dashed and dash-dotted black curves corresponding to the capacitance-corrected and uncorrected resonances, respectively. This conductance is later subtracted from the tunnelling data obtained when the wires are conducting, allowing us to separate the 'parasitic' from the 1D-2D tunnelling signal. Note that the quality of the fit with $d =$const. allows us to rule out a potential $d \equiv d(B)$ dependence due to the Lorentz repulsion between the wells, at least up to 7 T, which is the highest field used in our experiment.

upper well are approximately $\sim 100/70 = 1.4$ that of the lower well.

It is worth noting also that, particularly at high fields where the sign of k changes while tunnelling, one could expect the Lorentz force to act in such a way as to force carriers in each well further apart. This, in practice, would translate in having $d \equiv d(B)$. However, as can be seen from Fig. S5, no such correction is needed up to at least 7 T and so we can safely rule it out for the rest of our analysis.

1.6.3 1D-2D tunnelling signal

In the previous section we discussed how to constrain $c^{2\mathrm{D}}_{\mathrm{UW}}$, $c^{2\mathrm{D}}_{\mathrm{LW}}$, $m^*_{2\mathrm{D}}$, and d from the background data, *i.e.* using the conductance maps with the 1D wires past pinch-off. Since the observed modulation by the wire gate to the injection region is very small however, as can be seen by noting that the density under the parasitic and the wire regions closely match here (see Fig. S4C), it is reasonable to expect the same values to apply when fitting to the 2D signal even as the 1D channels are now conducting.

We map the 1D subbands as shown in Fig. S4A. Under the current geometry, most devices usually display between three to four subbands below the 2D band. By setting appropriate

values for $V_{\rm WG}$, we are able to map the dispersion of the system at different subband occupancy values (see an example in Fig. 4A in the main text). For now, we will restrict ourselves to the single-subband regime. From Fig. S4, both the Fermi energies as well as the 1D (2D) densities in the wire ('parasitic') region can be determined by using equations (S17), (S18) and (S19).

The procedure followed when fitting the tunnelling signal arising from the wire region is analogous to what was done for the 'parasitic' injection region. Since $c^{\rm 2D}_{\rm UW}$, $c^{\rm 2D}_{\rm LW}$ and d have already been obtained, the only parameters left to be determined are $B^{\rm w}_{-}$ and $B^{\rm w}_{+}$ (the zero-bias field intersects for the 1D signal, obtained similarly by visual inspection of the tunnelling maps), $m_{\rm UW} = m^{*}_{\rm 1D}$ (the effective mass of the 1D electrons in the upper well), and $c^{\rm 1D}_{\rm UW}$ and $c^{\rm 1D}_{\rm LW}$ (the capacitances per unit area in both wells). For the latter the values found, $c^{\rm 1D}_{\rm UW} = c^{\rm 1D}_{\rm LW} = 0.0026\,{\rm Fm}^{-2}$, were significantly lower that those predicted in COMSOL. Using the larger values for capacitance completely distorts the dispersions and cannot be accounted for by errors in other parameters as the constraints are strong. We interpret the observed deviations as a significantly stronger effect of interactions in 1D that push the band down, reducing the rate of filling the wire, $dn_{\rm 2D}/dE_{\rm F}$, or in other words, increasing the energy required to add an extra electron. This results in a decrease in the quantum capacitance $C_{\rm Q}$, therefore making the total capacitance C no longer solely determined by the geometric contributions as was done in our simulation, see (S26).

The effective mass of electrons in the lower well in the 1D-2D regime was found to be slightly higher than in the 2D-2D scenario ($\simeq 0.067m_{\rm e}$ vs $\simeq 0.062m_{\rm e}$) that we attribute to the different amount of interactions, which electrons in the Fermi liquid of the bottom layer experience when the wire-gate depletes significantly the upper layer. In other words, reduction in screening by the upper layer changes interactions and hence $m^{*}_{\rm 2D}$.

Model 1: Single Fermi Sea and Single Plasmon Let us first assume that there is a single, uniquely determined 1D effective mass, which matches the 2D non-interacting effective mass, $m^{*}_{\rm 1D} = m^{*}_{\rm 2D}$. From Fig. S6A it can be seen then that while the 1D mode is nicely fitted in the hole sector ($V_{\rm DC} < 0$), the same does not happen in the particle sector ($V_{\rm DC} > 0$), particularly at very high biases, even when correcting for capacitance. We therefore conclude that the 1D excitation cannot be fully captured by a single 1D effective mass.

Model 2: Single Fermi Sea and Two Plasmons We now assume that there are both spin- and charge-type excitations in our 1D system. We note that we have previously observed spin-charge separation in similar devices [9]. Under our configuration, we also know that the 1D excitations in the hole sector correspond to a spinon mode, while that in the particle sector to a holon mode [21]. Since each mode corresponds to a different kind of excitations, it is reasonable to expect different effective masses for each, which we label as $m_{\rm s}$ and $m_{\rm c}$ respectively. As before, it can be shown (see Fig. S6B) that while $m_{\rm s} = m^{*}_{\rm 2D}$ provides a good match to the data, even when taking $m_{\rm c} < m^{*}_{\rm 2D}$ one still fails to fully capture the observed behaviour for this mode. This is particularly noticeable when taking the ${\rm d}G/{\rm d}B$ differential. We also note that any fit to the holon branch in the particle sector must also catch the low-energy charge mode, that is, the holon branch in the hole sector, when extended back to it, which is clearly not the case.

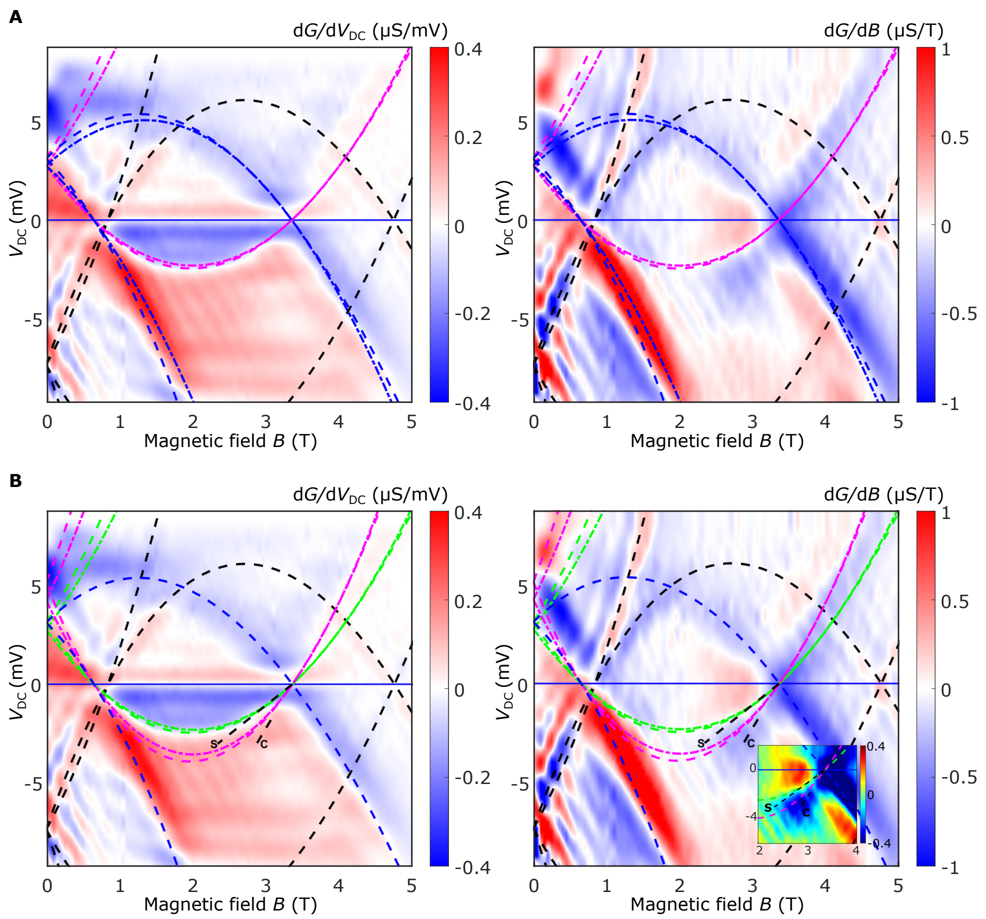

Figure S6: **Single-subband regime: One Fermi Sea.** Tunnelling-conductance differentials $\mathrm{d}G/\mathrm{d}V_{\mathrm{DC}}$ and $\mathrm{d}G/\mathrm{d}B$ as a function of the DC-bias V_{DC} and the in-plane magnetic field B, for a $1.7\,\mu$m device, mapped at $V_{\mathrm{WG}} = -0.515\,\mathrm{V}$. (**A**) Model 1, with a single plasmon type, where $m^*_{\mathrm{1D}} = m^*_{\mathrm{2D}} = 0.93\,m_{\mathrm{b}}$. The dashed black curves indicate the location of the subtracted 2D-2D 'parasitic' tunnelling signal mapped in Fig. S5. The dashed and dash-dotted magenta curves mark the capacitance corrected and uncorrected resonances arising from the tunnelling between the LW ground states and the UW wire region. They reveal the dispersion of the elementary excitations in the 1D UW wire region. Similarly, the dashed and dash-dotted blue curves mark the location of the resonances resulting from the reverse tunnelling process, between the UW ground states and the LW, revealing the dispersion of the 2D LW. (**B**) Same as in (**A**) but now fitted using Model 2, where two types of excitations, spin and charge, are allowed. The dashed black and blue lines represent the same dispersions as before. The green curves correspond to a spinon mode with $m_{\mathrm{s}} = m^*_{\mathrm{2D}}$. The magenta lines, on the other hand, mark a holon mode with $m_{\mathrm{c}} < m^*_{\mathrm{2D}}$. Inset: Spin-charge separation (dashed black 'S' and 'C' lines) near the $+k_{\mathrm{F}}$ point at low bias.

Model 3: Two Fermi Seas and Two Plasmons Based on the previous analyses we will now consider the spectroscopic predictions of the 1D Fermi-Hubbard model (see details in the

Supplementary Text 'Spin and charge excitations' above and Fig. S2C), where we now assume not only different effective masses m_s and m_c but also different densities, and therefore different Fermi momenta for both the spinon and the holon modes. We start from the assumption that the dispersion of both modes, f, is parabolic, before correcting for capacitance. That gives us

$$f_1(k) = \frac{\hbar^2 k^2}{2m_\mathrm{s}} - E_\mathrm{F}^\mathrm{s} = \frac{\hbar^2}{2m_\mathrm{s}}(k^2 - k_\mathrm{F}^2) \tag{S28}$$

and

$$f_2(k) = \frac{\hbar^2 (k + k_\mathrm{F})^2}{2m_\mathrm{c}} - E_\mathrm{F}^\mathrm{c} = \frac{\hbar^2}{2m_\mathrm{c}}\left[(k + k_\mathrm{F})^2 - 4k_\mathrm{F}^2\right] \tag{S29}$$

where E_F^s, m_s and E_F^c, m_c are the respective Fermi energies and the renormalised masses, with $k_\mathrm{F} \equiv k_\mathrm{F,UW}$ for simplicity. The charge mode, forming between $-3k_\mathrm{F}$ and $+k_\mathrm{F}$, has a density of states half that of the spinon excitation, between $\pm k_\mathrm{F}$. An analogous expression can also be obtained for the holon branch between $-k_\mathrm{F}$ and $+3k_\mathrm{F}$. Since $v = \mathrm{d}\omega/\mathrm{d}k = \hbar^{-1}\mathrm{d}E/\mathrm{d}k$, we get

$$v^\mathrm{s} = \frac{1}{\hbar}\frac{\mathrm{d}f_1}{\mathrm{d}k}\bigg|_{k=k_\mathrm{F}} = \frac{\hbar k}{m_\mathrm{s}}\bigg|_{k=k_\mathrm{F}} = \frac{\hbar k_\mathrm{F}}{m_\mathrm{s}} \tag{S30}$$

and

$$v^\mathrm{c} = \frac{1}{\hbar}\frac{\mathrm{d}f_2}{\mathrm{d}k}\bigg|_{k=k_\mathrm{F}} = \frac{\hbar (k + k_\mathrm{F})}{m_\mathrm{c}}\bigg|_{k=k_\mathrm{F}} = \frac{2\hbar k_\mathrm{F}}{m_\mathrm{c}}, \tag{S31}$$

therefore arriving at

$$\frac{v^\mathrm{c}}{v^\mathrm{s}} = \frac{K_\mathrm{s}}{K_\mathrm{c}} = \frac{2m_\mathrm{s}}{m_\mathrm{c}}. \tag{S32}$$

Equation (S32) allows us to relate the charge-to-spin velocity ratio with the phenomenological Luttinger parameters $K_\mathrm{c,s}$, which account for the renormalisation of the effective masses m_s and m_c due to the 1D confinement. We have $m_\mathrm{s} = m_\mathrm{b} K_\mathrm{s}$ and $m_\mathrm{c} = m_\mathrm{b} K_\mathrm{c}$, with $K_\mathrm{c} \neq K_\mathrm{s}$. The ratio $K_\mathrm{c}/K_\mathrm{s}$ is a good estimate of the interaction strength. Note also that the extra factor of two in the third term of (S32) arises from the assumption of different densities of states of the collective modes in each Fermi sea, or in other words because k_F is twice as large for holons as it is for spinons. For repulsive interactions, since $K_\mathrm{s} > 1$ and $K_\mathrm{c} < 1$, we have $m_\mathrm{c} < 2m_\mathrm{s}$.

In Fig. S7A we fit the data using the model with two Fermi seas. The dashed curves were obtained using the parabolic model described in this section while the open-circle lines correspond to solutions of the Fermi-Hubbard model. Both models are in very good agreement across all momentum and energy range experimentally probed. Note how, unlike before, the holon mode emanating from $+k_\mathrm{F}$ is now fully captured both in the hole and particle sectors. The line shapes at zero magnetic field for various tunnelling processes are also shown in Fig. S7B. The tunnelling resonance peak at large negative bias (black dashed line in Fig. S7A) corresponds to 2D-2D 'parasitic' tunnelling, while at positive bias the main contribution to conductance comes from both 1D and 2D processes. Unlike the 2D-2D peak however, some significant amount of broadening can be observed. We interpret this as the superposition of both the 2D (dashed

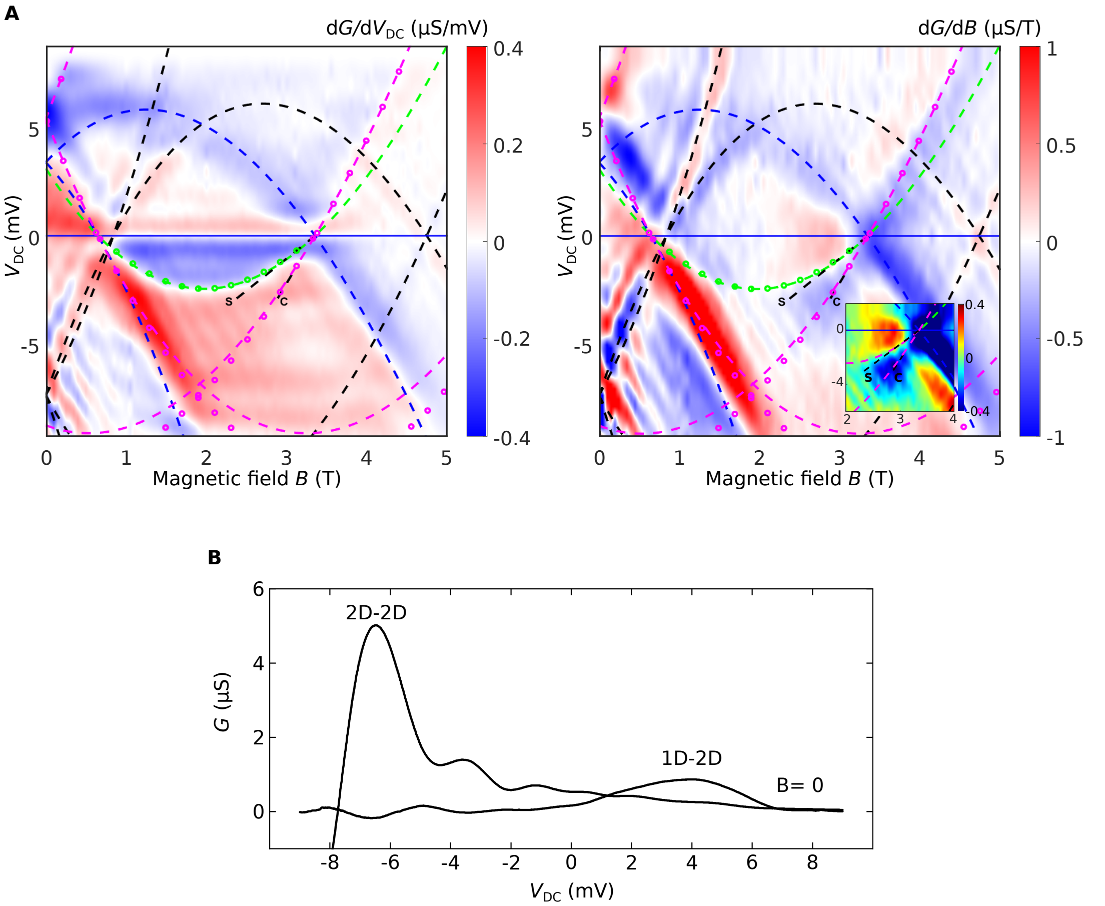

Figure S7: **Single-subband regime: Two Fermi Seas and Fermi-Hubbard Model.** (**A**) Tunnelling-conductance differentials $\mathrm{d}G/\mathrm{d}V_{\mathrm{DC}}$ and $\mathrm{d}G/\mathrm{d}B$ as a function of the DC-bias V_{DC} and the in-plane magnetic field B, for a 1.7 μm device, mapped at $V_{\mathrm{WG}} = -0.515\,\mathrm{V}$ and fitted using the model of two Fermi seas, see text. Dashed black and blue curves have the same meanings as before, see Fig. S6. The dashed green and magenta lines mark the 1D dispersions for spinons and holons, respectively. The open-circle curves mark the corresponding solutions of the Fermi-Hubbard model for $N = 54$. Inset: Spin-charge separation (dashed black ‘S’ and ‘C’ lines) near the $+k_{\mathrm{F}}$ point at low-bias. (**B**) Conductance G line-cuts at $B = 0$, showing the broadened 1D-2D tunnelling resonance peak, and the equivalent 2D-2D background-calibration trace with the wires pinched off. The broad 1D-2D peak comes from the overlap of the dashed blue and dashed magenta resonances as described in **A**, see section 1.6.4 for more details.

blue) and 1D (dashed magenta) dispersions, as predicted by our model, see the next section for discussion. Most importantly, however, this feature is completely missed by previous models, see Fig. S6.

1.6.4 Tunnelling resonances at zero field

Besides the full mapped dispersions, we can also inspect the various tunnelling processes seen at zero magnetic field (see Fig. S8A for a 5 μm device) to further constrain our model. The two panels show $\mathrm{d}G/\mathrm{d}V_{\mathrm{WG}}$ vs wire-gate voltage V_{WG} and DC bias V_{DC}, for two values of the voltage V_{PG} on a gate that allows us to move the 'parasitic' features around by changing the density in the 'parasitic' region. All features at negative DC bias move away for positive V_{PG}, indicating they are not related to the 1D wires themselves. They correspond to the 2D-2D 'parasitic' tunnelling discussed in section 1.6.2. On the other hand, at positive V_{DC}, the main contribution comes from the superposition of both 1D and 2D tunnelling happening in the wire region, which was discussed in section 1.6.3. Note that, not only are these features unaffected by V_{PG}, but they also disappear around $V_{\mathrm{WG}} = -0.6$ V, the same value at which the wires are known to pinch off, see Fig. 1C. Fig. S8B shows the line profiles of the data shown in Fig. S8A for positive V_{PG}. As can be seen, the tunnelling signal corresponding to the wires slowly broadens as the confinement is made progressively stronger (by making V_{WG} more negative, hence reducing the density).

At $B = 0$ there should be a peak in current as a function of V_{DC} as the concentric spectral functions of the 1D and 2D systems move in and out of alignment. The conductance will therefore have a peak and a trough (negative differential conductance, NDC), like that shown at small wire-gate voltages in Fig. S8B. Higher 1D subbands and the small overlap of a large number of states in the two systems (because at $B = 0$ their spectral functions are not offset in momentum), enhance the tunnelling above the peak, reducing the depth of the trough and moving the peak position towards higher bias. Nevertheless, as the wires are squeezed, from $V_{\mathrm{WG}} = -0.53$ V onwards, first a kink, then two well-defined features can be seen, unaffected by V_{PG} and therefore not arising from the parasitic region. While this extra feature could indicate tunnelling into an empty second 1D subband, there is no sign of this anywhere else in our full energy–momentum maps such as that in Fig. 2C. Also, the splitting is much too great to be explained by different effective masses of the 2D Fermi-liquid quasi-particles and the 1D spinon modes. Note also that, for the latter case, the spinon mode would be equivalent to that coming from the $+k_{\mathrm{F}}$ point, which we observe to be absent in the particle sector, and is also therefore not expected here below $-k_{\mathrm{F}}$.

The observed feature is separated from the main peak by about the same bias as the 2D (dashed blue) and 1D holon (dashed magenta) branches emanating from the $-k_{\mathrm{F}}$ point in the particle sector at $B = 0$. For each wire-gate voltage, we find good agreement between these zero-field features and the position of the 2D and 1D holon spectral modes as predicted from the full dispersion maps. This is completely missed by Models 1 and 2 previously discussed, and can only be successfully accounted for if two Fermi seas are assumed.

1.6.5 Multiple-subband occupancy

Every time a new subband starts being occupied, another four parameters (B_{-}^{w}, B_{+}^{w}, m_{s} and m_{c}) need to be added. The capacitances $c_{\mathrm{UW}}^{\mathrm{1D}}$ and $c_{\mathrm{LW}}^{\mathrm{1D}}$ also need to be updated, with their values now

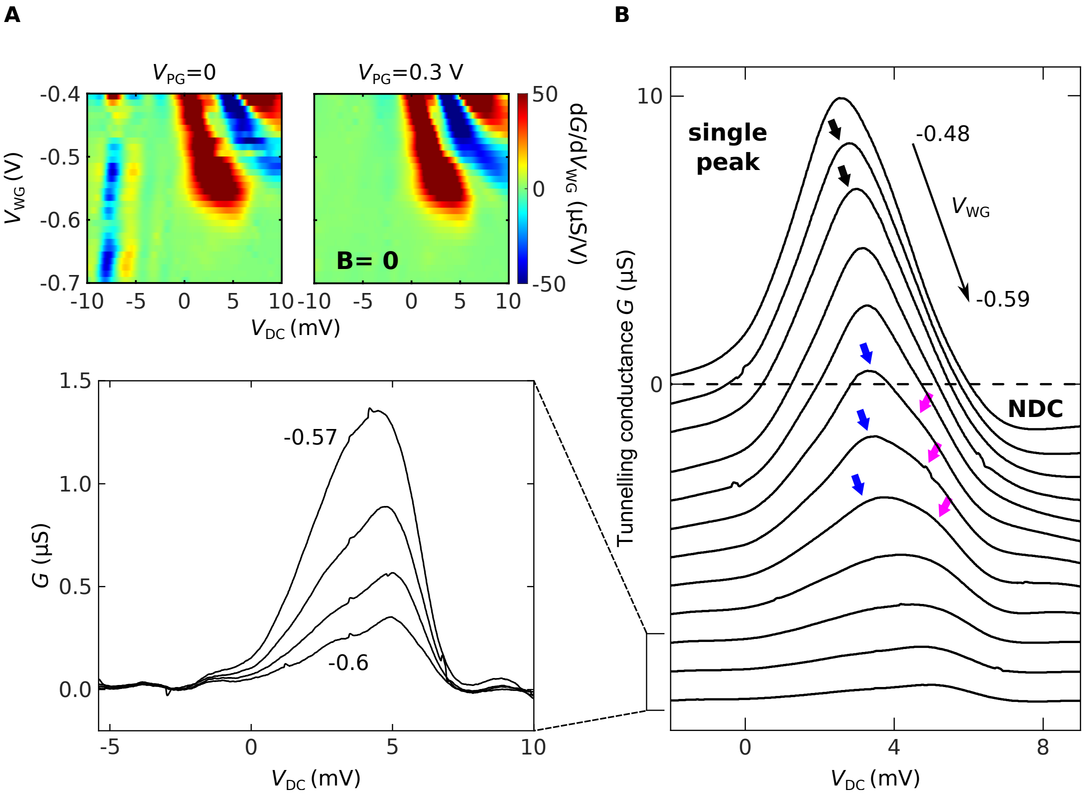

Figure S8: **Tunnelling processes at zero field.** (**A**) $\mathrm{d}G/\mathrm{d}V_{\mathrm{WG}}$ vs V_{WG} and V_{DC}, at $B = 0$ for both $V_{\mathrm{PG}} = 0$ and $V_{\mathrm{PG}} = 0.3\,\mathrm{V}$ (the latter moves the fringes arising in the 'parasitic' region out of the range of the plot). The conductance peak seen at $V_{\mathrm{DC}} > 0$ disappears at $V_{\mathrm{WG}} = -0.6\,\mathrm{V}$, the same value at which the wires pinch off in Fig. 1C; (**B**) Horizontal line-cuts of the conductance data shown in **A** for positive V_{PG}. Here, the signal can be seen to slowly broaden as V_{WG} is made progressively more negative (*i.e.*, by increasing the level of confinement in the wires and hence decreasing the density), eventually separating into two distinct features, with a similar spacing to that of the 2D and 1D holon modes in the particle sector. Every curve, except for $V_{\mathrm{WG}} = -0.48\,\mathrm{V}$, has been offset for clarity. The left-hand panel shows, without any offsets, the line-cuts for $V_{\mathrm{WG}} \leq -0.57\,\mathrm{V}$ (*i.e.*, as the 1D channels pinch off).

lying somewhere in between the single-subband 1D-2D and the 2D-2D regimes. This leads to, for example, a total of 6+4+4+4=18 parameters when in the three subband regime.

We have restricted our analysis, where applicable, to the bottom two subbands even when more are occupied, as it becomes progressively more difficult to accurately and reliably analyse the data, see Fig. S9 and Fig. 4 in the main text. One of the difficulties is that, as the number of subbands is increased, it becomes visually harder to extract B_{-}^{w} for most subbands. We know however that a high degree of symmetry exists between all 1D subbands (see Fig. S4A). Since the 1D channels have approximately parabolic confinement potentials, the subband dispersions

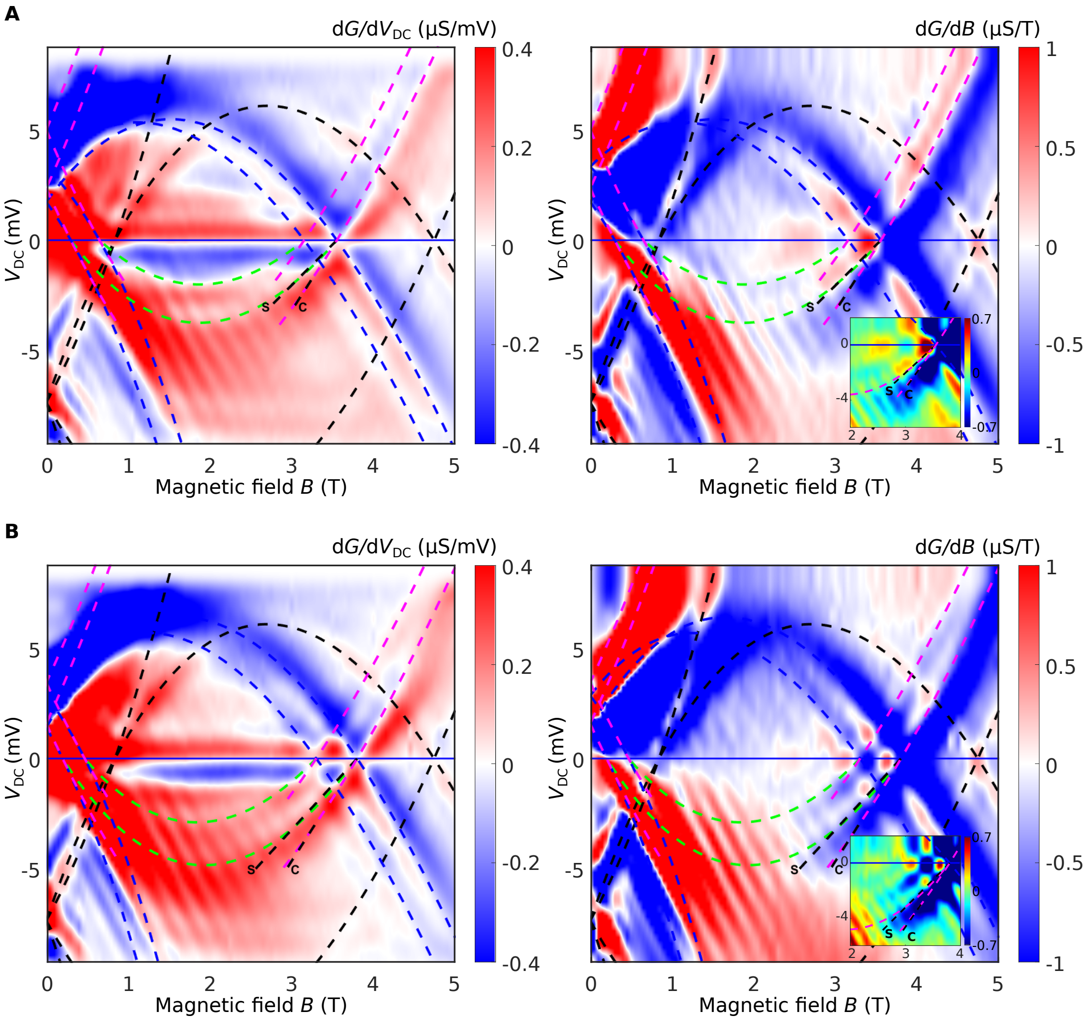

Figure S9: **Multiple-subband regime.** Tunnelling conductance differentials $\mathrm{d}G/\mathrm{d}V_{\mathrm{DC}}$ and $\mathrm{d}G/\mathrm{d}B$ plotted as a function of the DC-bias V_{DC} and the in-plane magnetic field B for a 1.7 μm device, mapped with (**A**) two ($V_{\mathrm{WG}} = -0.455\,\mathrm{V}$) and (**B**) three ($V_{\mathrm{WG}} = -0.405\,\mathrm{V}$) occupied subbands, respectively. The dashed coloured curves have the same meaning as before (see Fig. S6 and S7). Insets show spin-charge separation near the $+k_{\mathrm{F}}$ point for the bottom-most occupied subband.

should approximately follow that of a harmonic oscillator. Therefore, we have

$$\frac{B_{+,2} + B_{-,2}}{2} = \frac{B_{+,1} + B_{-,1}}{2}, \tag{S33}$$

where $B_{\pm,n}$ denote the crossing points between the nth-subband and the B axis at $V_{\mathrm{DC}} = 0$. Similarly, one can look at the crossing point $V_{0,\mathrm{n}}$ between the nth-subband and the V_{DC} axis at

$B = 0$, in which case we obtain

$$eV_{0,n} = \pm\frac{\hbar^2}{2m^\star}(k^2_{\text{F,UW},n} - k^2_{\text{F,LW}}) \Longrightarrow V_{0,n} = \pm\frac{ed^2}{2m^\star}B_{+,n}B_{-,n}. \tag{S34}$$

Here, $k_{\text{F,UW},n}$ refers to the Fermi wavevector of the spinon mode of the nth subband while $\pm$ labels the lower and upper layers, respectively. The second equality is obtained by converting k_Fs into $B_{\pm,n}$ using (S17). Combining both (S33) and (S34) we arrive at

$$\begin{aligned}(B_{+,2} - B_{-,2})^2 &= (B_{+,2}+B_{-,2})^2 - 4B_{+,2}B_{-,2} \\ &\Longrightarrow \frac{B_{+,2} - B_{-,2}}{2} = \sqrt{\left(\frac{B_{+,1} + B_{-,1}}{2}\right)^2 \pm V_{0,2}\frac{2m}{ed^2}}.\end{aligned} \tag{S35}$$

Equation (S35) can be used to determine the position of the second subband by varying $V_{0,2}$ and plotting the dispersions with the resultant $B_{\pm,2}$ until the best match is obtained. Since $B_{+,2}$ is usually well-observed in the data, this effectively imposes a very strong constraint on $B_{-,2}$.

Another difficulty of the multiple-occupancy regime has to do with extracting the holon and spinon masses, m_c and m_s. The first is usually obtained by looking at the holon branch emerging from $+k_\text{F}$ at high bias. However, as more subbands come into play these become harder to resolve separately. Similarly to what was done in the single-subband regime, we can obtain a second constraint on m_c by looking at the peak broadening at $B = 0$. On the other hand, m_s is extracted by matching to the spinon-mode dispersion in the hole sector. This is usually clearly observed for at least the bottom two subbands. Nevertheless, since the spin and charge velocities can only be obtained for the bottom-most occupied subband, this ratio cannot be compared to the independently obtained mass ratios, as was done when only one subband was occupied. Overall, this translates into a slight increase of the error with higher subband occupancy, as seen from Fig. 2E and 4 in the main text.

REFERENCES AND NOTES

1. T. Giamarchi, *Quantum Physics in One Dimension* (Clarendon Press, 2003).

2. S. Tomonaga, Remarks on Bloch's method of sound waves applied to many-fermion problems. *Prog. Theor. Phys.* **5**, 544–569 (1950).

3. J. M. Luttinger, An exactly soluble model of a many-fermion system. *J. Math. Phys.* **4**, 1154–1162 (1963).

4. F. D. M. Haldane, 'Luttinger liquid theory' of one-dimensional quantum fluids. I. Properties of the Luttinger model and their extension to the general 1D interacting spinless Fermi gas. *J. Phys. C Solid State Phys.* **14**, 2585–2609 (1981).

5. M. Bockrath, D. H. Cobden, J. Lu, A. G. Rinzler, R. E. Smalley, L. Balents, P. L. McEuen, Luttinger-liquid behaviour in carbon nanotubes. *Nature* **397**, 598–601 (1999).

6. H. Ishii, H. Kataura, H. Shiozawa, H. Yoshioka, H. Otsubo, Y. Takayama, T. Miyahara, S. Suzuki, Y. Achiba, M. Nakatake, T. Narimura, M. Higashiguchi, K. Shimada, H. Namatame, M. Taniguchi, Direct observation of Tomonaga-Luttinger-liquid state in carbon nanotubes at low temperatures. *Nature* **426**, 540–544 (2003).

7. Z. Shi, X. Hong, H. A. Bechtel, B. Zeng, M. C. Martin, K. Watanabe, T. Taniguchi, Y.-R. Shen, F. Wang, Observation of a Luttinger-liquid plasmon in metallic single-walled carbon nanotubes. *Nat. Photonics* **9**, 515–519 (2015).

8. O. M. Auslaender, H. Steinberg, A. Yacoby, Y. Tserkovnyak, B. I. Halperin, K. W. Baldwin, L. N. Pfeiffer, K. W. West, Spin-charge separation and localization in one dimension. *Science* **308**, 88–92 (2005).

9. Y. Jompol, C. J. B. Ford, J. P. Griffiths, I. Farrer, G. A. C. Jones, D. Anderson, D. A. Ritchie, T. W. Silk, A. J. Schofield, Probing spin-charge separation in a Tomonaga-Luttinger liquid. *Science* **325**, 597–601 (2009).

10. D. Laroche, G. Gervais, M. P. Lilly, J. L. Reno, Positive and negative Coulomb drag in vertically integrated one-dimensional quantum wires. *Nat. Nanotechnol.* **6**, 793–797 (2011).

11. B. J. Kim, H. Koh, E. Rotenberg, S.-J. Oh, H. Eisaki, N. Motoyama, S. Uchida, T. Tohyama, S. Maekawa, Z.-X. Shen, C. Kim, Distinct spinon and holon dispersions in photoemission spectral functions from one-dimensional $SrCuO_2$. *Nat. Phys.* **2**, 397–401 (2006).

12. J. Vijayan, P. Sompet, G. Salomon, J. Koepsell, S. Hirthe, A. Bohrdt, F. Grusdt, I. Bloch, C. Gross, Time-resolved observation of spin-charge deconfinement in fermionic Hubbard chains. *Science* **367**, 186–189 (2020).

13. G. Barak, H. Steinberg, L. N. Pfeiffer, K. W. West, L. Glazman, F. von Oppen, A. Yacoby, Interacting electrons in one dimension beyond the Luttinger-liquid limit. *Nat. Phys.* **6**, 489–493 (2010).

14. Y. Jin, O. Tsyplyatyev, M. Moreno, A. Anthore, W. K. Tan, J. P. Griffiths, I. Farrer, D. A. Ritchie, L. I. Glazman, A. J. Schofield, C. J. B. Ford, Momentum-dependent power law measured in an interacting quantum wire beyond the Luttinger limit. *Nat. Commun.* **10**, 2821 (2019).

15. S. Wang, S. Zhao, Z. Shi, F. Wu, Z. Zhao, L. Jiang, K. Watanabe, T. Taniguchi, A. Zettl, C. Zhou, F. Wang, Nonlinear Luttinger liquid plasmons in semiconducting single-walled carbon nanotubes. *Nat. Mater.* **19**, 986–991 (2020).

16. A. Imambekov, L. I. Glazman, Universal theory of nonlinear Luttinger liquids. *Science* **323**, 228–231 (2009).

17. T. L. Schmidt, A. Imambekov, L. I. Glazman, Fate of 1D spin-charge separation away from Fermi points. *Phys. Rev. Lett.* **104**, 116403 (2010).

18. T. L. Schmidt, A. Imambekov, L. I. Glazman, Spin-charge separation in one-dimensional fermion systems beyond luttinger liquid theory. *Phys. Rev. B* **82**, 245104 (2010).

19. O. Tsyplyatyev, A. J. Schofield, Y. Jin, M. Moreno, W. K. Tan, C. J. B. Ford, J. P. Griffiths, I. Farrer, G. A. C. Jones, D. A. Ritchie, Hierarchy of modes in an interacting one-dimensional system. *Phys. Rev. Lett.* **114**, 196401 (2015).

20. O. Tsyplyatyev, A. J. Schofield, Y. Jin, M. Moreno, W. K. Tan, A. S. Anirban, C. J. B. Ford, J. P. Griffiths, I. Farrer, G. A. C. Jones, D. A. Ritchie, Nature of the many-body excitations in a quantum wire: Theory and experiment. *Phys. Rev. B* **93**, 075147 (2016).

21. M. Moreno, C. J. B. Ford, Y. Jin, J. P. Griffiths, I. Farrer, G. A. C. Jones, D. A. Ritchie, O. Tsyplyatyev, A. J. Schofield, Nonlinear spectra of spinons and holons in short GaAs quantum wires. *Nat. Commun.* **7**, 12784 (2016).

22. E. H. Lieb, F. Y. Wu, Absence of Mott transition in an exact solution of the short-range, one-band model in one dimension. *Phys. Rev. Lett.* **20**, 1445–1448 (1968).

23. Y. Jin, M. Moreno, P. M. T. Vianez, W. K. Tan, J. P. Griffiths, I. Farrer, D. A. Ritchie, C. J. B. Ford, Microscopic metallic air-bridge arrays for connecting quantum devices. *Appl. Phys. Lett.* **118**, 162108 (2021).

24. H. J. Schulz, Correlation exponents and the metal-insulator transition in the one-dimensional hubbard model. *Phys. Rev. Lett.* **64**, 2831–2834 (1990).

25. H. Frahm, V. E. Korepin, Critical exponents for the one-dimensional Hubbard model. *Phys. Rev. B* **42**, 10553–10565 (1990).

26. V. Meden, K. Schönhammer, Spectral functions for the Tomonaga-Luttinger model. *Phys. Rev. B* **46**, 15753–15760 (1992).

27. J. Voit, Charge-spin separation and the spectral properties of Luttinger liquids. *Phys. Rev. B* **47**, 6740–6743 (1993).

28. H. Benthien, F. Gebhard, E. Jeckelmann, Spectral function of the one-dimensional hubbard model away from half filling. *Phys. Rev. Lett.* **92**, 256401 (2004).

29. G. D. Mahan, *Many-Particle Physics* (Plenum, 1990).

30. B. Lake, D. A. Tennant, C. D. Frost, S. E. Nagler, Quantum criticality and universal scaling of a quantum antiferromagnet. *Nat. Mater.* **4**, 329–334 (2005).

31. M. Mourigal, M. Enderle, A. Klöpperpieper, J.-S. Caux, A. Stunault, H. M. Rønnow, Fractional spinon excitations in the quantum Heisenberg antiferromagnetic chain. *Nat. Phys.* **9**, 435–441 (2013).

32. B. Lake, D. A. Tennant, J.-S. Caux, T. Barthel, U. Schollwöck, S. E. Nagler, C. D. Frost, Multispinon continua at zero and finite temperature in a near-ideal Heisenberg chain. *Phys. Rev. Lett.* **111**, 137205 (2013).

33. J.-S. Caux, J. M. Maillet, Computation of dynamical correlation functions of Heisenberg chains in a magnetic field. *Phys. Rev. Lett.* **95**, 077201 (2005).

34. F. Göhmann, A. Klümper, A. Seel, Integral representations for correlation functions of the XXZ chain at finite temperature. *J. Phys. A* **37**, 7625–7651 (2004).

35. A. Imambekov, T. L. Schmidt, L. I. Glazman, One-dimensional quantum liquids: Beyond the Luttinger liquid paradigm. *Rev. Mod. Phys.* **84**, 1253–1306 (2012).

36. F. H. L. Essler, Threshold singularities in the one-dimensional Hubbard model. *Phys. Rev. B* **81**, 205120 (2010).

37. O. Tsyplyatyev, A. J. Schofield, Spectral-edge mode in interacting one-dimensional systems. *Phys. Rev. B* **90**, 014309 (2014).

38. L. I. Glazman, I. M. Ruzin, B. I. Shklovskii, Quantum transport and pinning of a one-dimensional Wigner crystal. *Phys. Rev. B* **45**, 8454–8463 (1992).

39. M. Kim, S. G. Xu, A. I. Berdyugin, A. Principi, S. Slizovskiy, N. Xin, P. Kumaravadivel, W. Kuang, M. Hamer, R. K. Kumar, R. V. Gorbachev, K. Watanabe, T. Taniguchi, I. V.

Grigorieva, V. I. Fal'ko, M. Polini, A. K. Geim, Control of electron-electron interaction in graphene by proximity screening. *Nat. Commun.* **11**, 2339 (2020).

40. M. Gaudin, Un systeme a une dimension de fermions en interaction. *Phys. Lett. A* **24**, 55–56 (1967).

41. C. N. Yang, Some exact results for the many-body problem in one dimension with repulsive delta-function interaction. *Phys. Rev. Lett.* **19**, 1312–1315 (1967).

42. R. Orbach, Linear antiferromagnetic chain with anisotropic coupling. *Phys. Rev.* **112**, 309–316 (1958).

43. M. Ogata, H. Shiba, Bethe-ansatz wave function, momentum distribution, and spin correlation in the one-dimensional strongly correlated Hubbard model. *Phys. Rev. B* **41**, 2326–2338 (1990).

44. M. Gaudin, *The Bethe Wavefunction* (Cambridge Press, 2014).

45. C. F. Coll III, Excitation spectrum of the one-dimensional Hubbard model. *Phys. Rev. B* **9**, 2150–2158 (1974).

46. P. Vianez, O. Tsyplyatyev, C. Ford, Chapter Three - Semiconductor nanodevices as a probe of strong electron correlations, in *Frontiers of Nanoscience*, D. A. Ritchie, Ed. (Elsevier, 2021), vol. 20 of *Semiconductor Nanodevices*, pp. 31–66.

47. S. E. Laux, D. J. Frank, F. Stern, Quasi-one-dimensional electron states in a split-gate GaAs/AlGaAs heterostructure. *Surf. Sci.* **196**, 101–106 (1988).

48. E. Owen, C. Barnes, Ground-state electronic structure of quasi-one-dimensional wires in semiconductor heterostructures. *Phys. Rev. Appl.* **6**, 054007 (2016).

49. S. Luryi, Quantum capacitance devices. *Appl. Phys. Lett.* **52**, 501–503 (1988).

50. P. T. Coleridge, M. Hayne, P. Zawadzki, A. S. Sachrajda, Effective masses in high-mobility 2D electron gas structures. *Surf. Sci.* **361-362**, 560–563 (1996).

51. M. Hayne, A. Usher, J. J. Harris, C. T. Foxon, Exchange enhancement of the Landau-level separation for two-dimensional electrons in GaAs/$Ga_{1-x}Al_xAs$ heterojunctions. *Phys. Rev. B* **46**, 9515–9519 (1992).

52. A. T. Hatke, M. A. Zudov, J. D. Watson, M. J. Manfra, L. N. Pfeiffer, K. W. West, Evidence for effective mass reduction in GaAs/AlGaAs quantum wells. *Phys. Rev. B* **87**, 161307 (2013).

53. Y.-W. Tan, J. Zhu, H. L. Stormer, L. N. Pfeiffer, K. W. Baldwin, K. W. West, Measurements of the density-dependent many-body electron mass in two dimensional GaAs/AlGaAs heterostructures. *Phys. Rev. Lett.* **94**, 016405 (2005).

54. Y. Kwon, D. M. Ceperley, R. M. Martin, Quantum Monte Carlo calculation of the Fermi-liquid parameters in the two-dimensional electron gas. *Phys. Rev. B* **50**, 1684–1694 (1994).
